# A global perspective on the genomics of Moraxella catarrhalis

**DOI:** 10.1099/mgen.0.001488

**Published:** 2025-08-22

**Authors:** Makarena Gonzalez-Reyes, Ignacio Ramos-Tapia, Juan A. Ugalde

**Affiliations:** 1 Center for Bioinformatics and Integrative Biology, Facultad de Ciencias de la Vida, Universidad Andrés Bello, República 330, Santiago, Chile

**Keywords:** *Moraxella catarrhalis*, phylogenomics, phylogroup, pangenome

## Abstract

*Moraxella catarrhalis* is an opportunistic pathogen of the human respiratory tract, primarily associated with otitis media in children and exacerbations of chronic obstructive pulmonary disease in adults. Despite its clinical importance, the genomic diversity and functional specialization of *M. catarrhalis* remain insufficiently characterized. This study aimed to analyse the global genetic diversity of *M. catarrhalis* using whole-genome sequencing to identify phylogenetic lineages, antimicrobial resistance patterns and key virulence factors. Phylogenomic analysis of 345 publicly available genomes identified 3 phylogroups, of which 1 exhibited significant genomic divergence and was excluded from further analyses due to its potential classification as a separate species. The remaining two phylogroups corresponded to previously described seroresistant and serosensitive lineages. Phylogroup B exhibited a higher prevalence of antimicrobial resistance genes, particularly *bro-1* and *bro-2*, while phylogroup A exhibited unique metabolic adaptation, including genes encoding for the DppB-DppC-DppD dipeptide transport system. Both phylogroups shared crucial virulence factors, including UspA1 and UspA2, which facilitate adhesion and immune evasion. Potential therapeutic targets were identified, including PilQ, essential for type IV pilus biogenesis, and CopB, which plays a key role in iron acquisition and immune evasion. Overall, these findings highlight the significance of phylogenomics approaches in elucidating the genetic mechanisms underlying pathogenicity and resistance in *M. catarrhalis*, providing insights for future therapeutic and preventive strategies.

**Impact Statement**

This study presents a comprehensive phylogenomic analysis of *Moraxella catarrhalis*, expanding current knowledge of its genetic diversity, antimicrobial resistance and virulence factors. By analysing 345 global genomes, we identified 3 distinct phylogroups, 1 of which shows substantial genomic divergence, suggesting it may represent a novel species within the *Moraxella* genus. Our findings confirm the existence of seroresistant and serosensitive lineages, providing new insights into their metabolic adaptations and differential antibiotic resistance profiles. Notably, phylogroup B harbours a higher prevalence of *β*-lactam resistance genes, while phylogroup A exhibits unique peptide transport systems. This work reinforces the evidence that phylogenomic approaches are crucial for understanding the evolutionary dynamics and pathogenic potential of *M. catarrhalis*. The identification of conserved virulence factors and potential therapeutic targets, such as PilQ and CopB, highlights the significance of this pathogen in respiratory infections and underscores the need for targeted interventions. Our results not only clarify the population structure of *M. catarrhalis* but also provide a valuable genomic framework for future studies on antimicrobial resistance and vaccine development, with broad utility for microbiologists, clinicians and public health researchers.

## Data Summary

The authors confirm that all supporting data, code and protocols have been provided within the article or through supplementary data files.

## Introduction

*Moraxella catarrhalis* is a Gram-negative bacterium from the order *Pseudomonadales* that commonly resides in the upper and lower respiratory tract of humans [1]. *M. catarrhalis* can act as an opportunistic pathogen depending on several factors, such as age, being more prevalent in infants and the elderly [2], immune status [3] and the presence of underlying chronic respiratory diseases such as chronic obstructive pulmonary disease (COPD), chronic bronchitis and bronchiectasis [4]. In the upper respiratory tract, this bacterium is one of the leading causes of otitis media, alongside *Streptococcus pneumoniae* and *Haemophilus influenzae* [3,4], accounting for 15–20% of acute otitis media episodes in children [5,7]. Additionally, it is responsible for ~20% of acute bacterial sinusitis cases in children and a smaller proportion in adults [7,9]. In the lower respiratory tract, *M. catarrhalis* is a major aetiological agent of infections, such as pneumonia and exacerbations of COPD, in adults, causing an estimated 2–4 million cases annually in the USA, which accounts for ~10% of all exacerbations [7,10]. In immunocompromised individuals, *M. catarrhalis* can lead to severe infections, including septicaemia, meningitis and endocarditis [11,14]. Furthermore, hospital outbreaks of respiratory diseases associated with this micro-organism have been reported [14,16], underscoring its clinical and epidemiological significance in both community and healthcare settings.

Previous studies using molecular typing methods, including multilocus sequence typing (MLST), 16S and 23S rRNA gene sequencing, fingerprinting of outer membrane proteins and restriction fragment length polymorphism [17,21], have suggested that *M. cattarhalis* is composed of two distinct lineages, seroresistant (SR) and serosensitive (SS), with different virulence potentials [19]. In addition, divergent strains with limited genetic homology to these lineages have also been described [21]. The SR lineage, primarily associated with 16S ribotype (RB) 1 strains [17,19], exhibits a highly pathogenic profile due to two key virulence traits: high resistance to the human complement system and efficient adhesion to respiratory epithelial cells [21]. This lineage is more frequently associated with respiratory tract diseases, as 51% of isolates are derived from individuals with diseased respiratory tracts [19,22]. In contrast, the SS lineage, composed of strains with RB2 and RB3 ribotypes [17,19], is sensitive to complement-mediated killing and exhibits lower adhesion efficiency, being less commonly associated with disease (15% of isolates from diseased individuals) [19,22]. Beyond these molecular classifications, whole-genome comparative analyses have confirmed that the SR and SS lineages are evolutionarily distinct. These analyses revealed an average nucleotide identity (ANI) of 95.78% between the two lineages, as well as differences in genome size and the composition of their respective supragenomes, with ~12.4% of the combined gene repertoire being non-shared [20]. Despite these differences, both lineages retain a conserved set of essential genes involved in fundamental cellular processes, supporting their classification within the same species. However, their independent evolutionary trajectories indicate that they diverged from a common ancestor and evolved separately [19], reflecting their genetic and adaptive complexity.

*M. catarrhalis* has developed several strategies to evade antibiotic action, primarily through three mechanisms: the production of *β*-lactamases, efflux pumps and biofilm formation. The primary resistance strategy employed by *M. catarrhalis* involves the production of *β*-lactamase enzymes [23], specifically the *BRO-1* and *BRO-2* variants, which can inactivate penicillin and other *β*-lactam antibiotics [24]. Currently, more than 95% of clinical isolates exhibit resistance to penicillin [25,26]. In addition to *β*-lactamase production, *M. catarrhalis* possesses resistance-nodulation-division-type efflux pumps, specifically AcrAB-OprM, which confer intrinsic resistance against multiple classes of antimicrobials, including *β*-lactams, quinolones and aminoglycosides [27,28]. These resistance mechanisms can be activated by environmental stress, such as a cold shock at 26 °C, or by exposure to antibiotics themselves, thus enhancing antibiotic resistance through active expulsion of the compounds [28,29]. Another critical strategy used by *M. catarrhalis* to resist antibiotic action is biofilm formation. Biofilms are bacterial communities that adhere to surfaces and are embedded in an extracellular matrix, which acts as a physical barrier against antimicrobial agents [30,31]. Biofilm formation is directly associated with persistent infections, such as chronic otitis media, where *M. catarrhalis* has been detected alongside other respiratory pathogens within biofilms [30]. Bacteria within these biofilms exhibit altered metabolism and reduced susceptibility to antibiotics, while maintaining conventional resistance mechanisms such as *β*-lactamase production [32,33].

Among the factors contributing to the pathogenesis of *M. catarrhalis*, the lipooligosaccharide (LOS) and several outer membrane proteins (OMPs) play a pivotal role [34,35], with the ubiquitous surface protein A (UspA) being a key element [35]. UspA is a high-molecular-weight, multifunctional trimeric adhesin abundantly expressed on the bacterial surface [36,38], where it facilitates adherence and evasion of the host immune system [39,40]. In the *M. catarrhalis* population, three genes encoding UspA variants – *uspA1*, *uspA2* and *uspA2H* – have been identified. Clinical studies indicate that *uspA1* is present in 99% of isolates [41], while *uspA2* or *uspA2H* are expressed in a ratio of ~4:1 [41,42]. In terms of pathogenicity, UspA1 and UspA2H are primarily associated with biofilm formation and adherence to respiratory tract epithelial cells [34,43,45], facilitating bacterial colonization. On the other hand, UspA2 and, in some cases, UspA2H, contribute to serum resistance by binding host complement inhibitors, such as vitronectin and C4b-binding protein [46,47].

The clinical and epidemiological relevance of *M. catarrhalis* lies in its remarkable adaptability, antimicrobial resistance and genetic diversification. However, despite advances in its genomic characterization, a clear genetic basis explaining the differences in virulence among its lineages has not been identified. Previous genomic studies of *M. catarrhalis* have primarily focused on characterizing genetic diversity, identifying virulence factors and exploring phylogenetic relationships using MLST or limited genome sets. These efforts have revealed significant evolutionary complexity but have failed to establish consistent links between lineages, virulence factors and metadata associated with each strain, such as the year of collection, clinical presentation and geographic location [20,48, 49]. To address these limitations, it is essential to employ a genome-based comparative approach that captures the genetic diversity of the species. This approach enables the examination of how the genetic variability of *M. catarrhalis* affects virulence, antimicrobial resistance and other phenotypic traits. Using publicly available genomic data, this study aims to correlate phylogenetic lineages with key genomic characteristics, thereby providing new insights into the determinants of pathogenicity in this species.

## Methodology

### *M. catarrhalis* genome acquisition

A total of 217 public genomes [50] and 3,135 Sequence Read Archive (SRA) [48] files were retrieved from the National Center for Biotechnology Information (NCBI). The search was conducted using the term ‘*Moraxella catarrhalis*’, and the data were retrieved on 30 March 2024. According to the available metadata, the samples were collected between 1932 and 2024. The SRA reads were cleaned using fastp v0.23.4 [49,51], applying a minimum read length of 50 bp and allowing a maximum of 5 ambiguous bases per read. Additionally, a minimum Phred quality score of 15 was set, along with a maximum of 40% unqualified bases per read. Subsequently, the reads were evaluated to determine if they correspond to *M. catarrhalis* using Kraken2 v2.1.3 [52,53], with the Standard database, which includes bacteria, archaea, human, plasmid, UniVec_Core and viral, applying a selection threshold ≥98% for the percentage of fragments covered by the clade rooted in a taxon, ensuring a reliable identification of the micro-organism. To determine whether any reads matched previously downloaded genomes, alignments were performed between the reads and the genomes. A read was considered distinct from the assembled genomes if the percentage of mapped reads and properly paired reads was ≤98 %, and the number of singleton reads (unpaired reads) was ≥0.02%. The reads were assembled using *SPAdes* v3.15.5 [54], and the quality of both the assemblies and the previously downloaded genomes was verified using CheckM2 v1.0.1 [55], ensuring a completeness ≥98% and a contamination level ≤2 %, according to the criteria published by Chklovski *et al*. [55]. MLST was performed using the software mlst v.23.0 [56,58], assigning alleles to the loci *abcZ*, *adk*, *efp*, *fumC*, *glyBeta*, *mutY*, *ppa* and *trpE* [59]. After applying the filters, a total of 345 genomes were kept for further analysis (Table S1, available with the online Supplementary Material).

### Phylogenomic analysis

Gene prediction and annotation were performed using Bakta v1.8.1 [60]. The files generated in this process served as the basis for the pangenome analysis conducted with Panaroo v1.3.3 [61], with a 100% threshold applied to the core genome. Core genes were aligned using MAFFT with default parameters [62]. Phylogenetic reconstruction was performed using RAxML v8.2.12 [63,66], with a GTR+CAT model. To validate the robustness of the phylogenetic relationships, support values were calculated from 1,000 bootstrap replicates. Phylogenetic clustering was performed using TreeCluster v1.0.4 [66,67], employing the single-linkage clustering method, which groups clusters based on the shortest pairwise distance between elements of different clusters [68], with a branch length threshold of 0.002.

### Pangenome analysis

Based on the gene presence–absence matrix generated by Panaroo v1.3.3 [61], the genes were classified into two main categories: core genes, present in 100% of the strains, and accessory genes, which are further subdivided into soft-core genes (95%≤strains<100 %), shell genes (15%≤strains<95 %) and cloud genes (strains<15 %) [69]. Subsequently, using the same matrix, Heap’s Law was calculated [70,71], which describes the relationship between the number of genomes analysed and the number of unique genes identified in the pangenome, determining the growth exponent (*γ*), a value that is used to classify the pangenome as closed if *γ*<0, open if *γ*>0 or reaching a fixed size (neither open nor closed) if *γ*=0 [70].

### Functional ontology assignments

For the functional ontology assignment, STRING v12.0 was used, which implements Gene Ontology (GO) as a classification system for gene-set enrichment analysis [72]. The analysis was performed using the amino acid sequences obtained from Bakta v1.8.1 [60], considering *M. catarrhalis* as the organism. The Benjamini and Hochberg correction was applied to adjust *P*-values and control the false discovery rate (FDR) [73], setting a significance level of 0.05. The analysis considered the biological process, molecular function and cellular component categories of GO.

### Antibiotic resistance

AMRFinderPlus v3.12.8 [73,75] was used to identify antibiotic and heavy metal resistance genes in plus mode, enabling the detection of additional genes related to resistance to biocides, metals and other stress response mechanisms. In addition, a specific search was conducted for the *bro-1* and *bro-2* genes, which are associated with *β*-lactam resistance in *M. catarrhalis*.

### Virulence factors

For the identification of proteins associated with virulence factors in *M. catarrhalis*, a total of 21 proteins described in the literature were selected, linked to key functions in adhesion, immune system evasion and iron acquisition. These included outer membrane proteins such as CopB (AAU43876.1/AAU43878.1/AAU43879.1) [76,78], LbpB (AAC31373.1) [76,78, 79], M35 (AAX99225.1) [76,78, 80], OmpCD (AAS75593.1) [76,78, 81], OmpE (AAA64436.1) [76,78, 82], OmpG1a (AAQ24464.1) [76,78, 83] and OmpG1b (AAS21221.1/AAS21227.1/AAS21232.1) [76,78, 84]; adhesion factors such as Mcap (ABM05621.1/ABM05625.1/ABM05628.1) [76,78, 85], MclS (AGM39706.1) [76,78, 86], McmA (ABL74969.1) [76,78], MhaB1 (ABQ43330.1/ABQ43331.1) [76,78, 87], MhaB2 (ABQ43328.1/ABQ43329.1) [76,78, 87], MhaC (ABQ42353.1/ABQ42358.1) [76,78, 87], MID/Hag (AAL78284.1/AAL78285.2/AAX56610.1) [76,78, 87, 88], UspA1 (AAD43469.1/AAF36416.1/AAN84895.1/ACC44784.1) [76,78, 89], UspA2 (AAD43468.1/AAF40119.1/AAN84896.1/AAO59378.1/AAW62383.1) [76,78, 89] and UspA2H (AAF40120.1/AAF40121.1/AAO59379.1/ABH07416.1) [76,78, 90]; iron acquisition systems such as TbpB (AAC34274.1/AAC34279.1) [76,78, 87, 91]; and proteins associated with type IV pilus formation such as PilA (AAV33390.1/AEB33767.1) [76,78, 92], PilQ (AAV33391.1) [76,78, 92] and PilT (AAV33392.1) [76,78, 92]. The sequences of these proteins were obtained from NCBI (https://www.ncbi.nlm.nih.gov/protein/) and used as queries in a blastp v2.14.0 [93] analysis against the amino acid sequences of the coding sequences (CDS) and small open reading frames (sORFs) generated by Bakta [60]. The search was performed on the proteins encoded by each assembled genome, retaining only the single best match per genome. Matches were prioritized first by *e*-value, followed by bitscore, percentage identity and finally percentage coverage of the alignment. Only hits with identity and alignment coverage equal to or greater than 80% were considered for downstream analysis [94,95].

### *In silico* classification of a species

To estimate the genomic relationship among the 345 sequences, we use the ANI between genome pairs in Pyani v0.2.12 [96], applying the ANIm method, which utilizes MUMmer for sequence alignment [97]. A threshold of ≥95% sequence identity by ANI was considered to determine that two genomes belong to the same species, as this value corresponds to the 70% identity threshold by DNA–DNA hybridization (DDH), traditionally used for the classification of prokaryotic species [96]. Additionally, *in silico* digital DNA–DNA hybridization (dDDH) [94,98,102] was applied to estimate the genomic relationship at the phylogroup level. This method is based on the same principle as experimental DDH, which is used for defining bacterial species. However, in this case, dDDH is *in silico*, implemented using assembled sequences. A threshold of ≥70% dDDH, along with a G+C content difference ≤1 %, was established to indicate that two strains belong to the same species [103]. Using the species identified from the dDDH analysis, a phylogenomic analysis was performed using the same procedure previously described, applying a core genome threshold of 80%. Subsequently, a new ANI calculation was conducted using Pyani v0.2.12 [96], aiming to assess the genomic relationship between these species and those within the phylogroup, thereby determining their genomic proximity.

## Results and discussion

A total of 217 public genomes and 3,135 SRA datasets of *M. catarrhalis* were retrieved from the NCBI. After applying quality control filters, a total of 345 genomes were retained for analysis, comprising 205 from assembled genomes and 140 from SRA data. According to available metadata, these genomes were collected between 1932 and 2024, spanning samples from at least nine countries, including Taiwan, Sweden, the Netherlands, Poland, the UK, Denmark, the USA, Australia and Chile. Host metadata revealed that the samples were derived from individuals with respiratory conditions, including otitis media, pneumonia, asthma and COPD.

### Pangenome composition and adaptive potential of *M. catarrhalis*

The pangenome analysis of *M. catarrhalis* revealed a total of 3,692 genes, which were classified based on their frequency in the analysed genomes. Of these, 1,061 genes were identified as core genes, present in 100% of the genomes. In general, these genes are involved in basic cellular and metabolic processes, growth and development, which are essential for the survival of the species [104]. On the other hand, 2,631 genes were classified as accessory genes, subdivided into 397 soft-core genes, 472 shell genes and 1,762 cloud genes. The shell and cloud genes contribute to genetic diversity and the adaptive capacity of isolates, including environmental adaptation, drug resistance and host adaptation [105,106]. In general, accessory genes are involved in secondary metabolism, stress response and interactions with other organisms and are likely associated with adaptation to specific environments [104]. The high number of accessory genes in the *M. catarrhalis* pangenome suggests significant genetic and adaptive variability in this species [107]. To assess the pangenome expansion dynamics, Heap’s Law was applied, yielding a value of *γ*=0.126. This result suggests that *M. catarrhalis* possesses an open pangenome, indicating a high capacity for acquiring new genes, possibly through horizontal gene transfer and the presence of various mobile genetic elements (MGEs) [108,109]. This capacity enables the species to adapt to different environments and respond to diverse selective pressures [110]. In the case of *M. catarrhalis*, the openness of its pangenome may reflect its genomic plasticity, potentially contributing to its adaptation to the human respiratory tract and its exposure to selective pressures such as antibiotics or the host immune response. However, further investigation is needed to determine whether these accessory genes are associated with MGEs, their distribution among phylogroups and how this pangenome compares to that of non-pathogenic *Moraxella* species.

### Phylogenetic clustering and lineage associations in *M. catarrhalis*

**Fig. 1.**
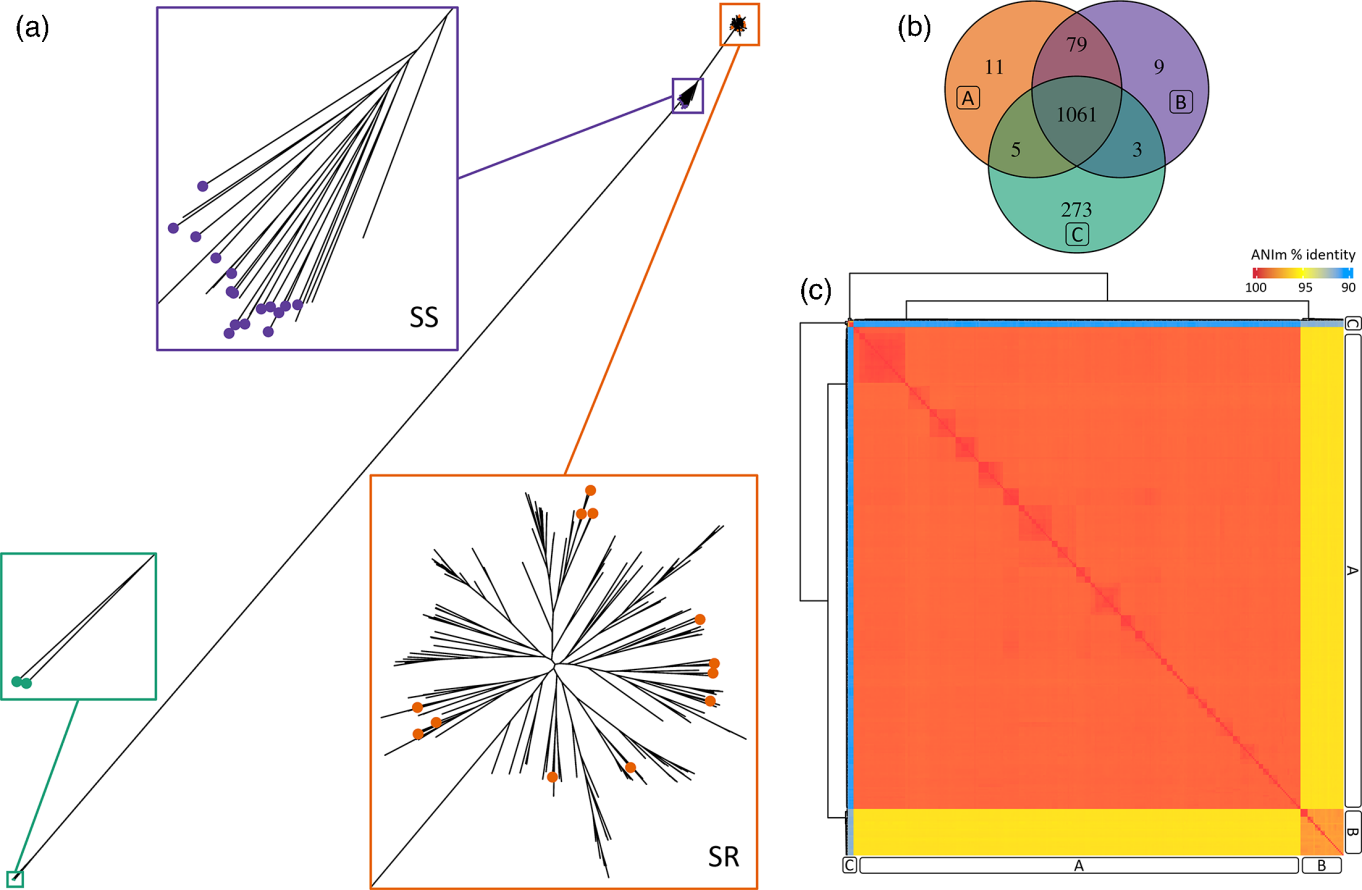
Phylogenetic and comparative genomic analysis of *M. catarrhalis*. (**a**) Maximum likelihood phylogenetic tree of *M. catarrhalis* strains, showing the three phylogroups: A (orange), B (purple) and C (green). Each dot on the branches represents a serotype evaluated experimentally. (**b**) Venn diagram displaying the number of unique and shared genes among phylogroups A, B and C. (**c**) Heatmap of ANI values calculated using Pyani, illustrating the genomic similarity among strains from different phylogroups. The letters A, B and C correspond to the classification of phylogroups: A represents the SR lineage, B the SS lineage and C a divergent lineage.

Through phylogenetic clustering, three distinct phylogroups were identified, designated as phylogroup A, phylogroup B and phylogroup C. To assess the possible correlation of these phylogroups with previously reported lineages, marker strains were used that had already been classified into specific lineages in previous studies [19]. The results indicated that phylogroup A was associated with a SR lineage, whereas phylogroup B corresponded to a SS lineage and phylogroup C to a divergent lineage (Fig. 1a). Although phylogroups A and B form distinct and well-supported clades, the bootstrap support at the node connecting both groups is relatively low, reflecting uncertainty in the deeper branching order rather than in the existence or coherence of each phylogroup. Additionally, an analysis of accessory genes was performed to evaluate their clustering structure (Figure S1). The results showed that the observed grouping matched the phylogroups defined in the core genome-based phylogeny, suggesting that both core and accessory genes reflect consistent patterns of diversification in *M. catarrhalis*.

### Genomic diversity and functional specialization among *M. catarrhalis* phylogroups

The analysis of the genes shared among the phylogroups (Fig. 1b) of *M. catarrhalis* revealed the presence of several essential genes involved in DNA repair, replication and structural integrity. Among the genes related to DNA recombination repair, *recA*, *recX*, *recC*, *recF*, *recG*, *recO* and *recR* were identified, playing a key role in post-replicative repair [111]. RecA is essential for homologous recombination and the response to RecFOR DNA damage [112,113], facilitating the restoration of stalled replication forks due to unrepaired lesions [114]. In *Escherichia coli*, the RecFOR and RecBCD pathways enable *RecA* loading in damaged regions [115,116], while in *Bacillus subtilis*, *RecO* and *RecR* are crucial for the formation of repair centres [117,118]. In addition, the genes *dnaA*, *dnaE*, *dnaK*, *dnaN* and *dnaQ* were identified as being involved in the initiation and elongation of DNA replication. *DnaA* is responsible for recognizing and unwinding the origin of replication (*oriC*), facilitating the loading of the *DnaB* helicase, in complex with *DnaC*, to establish the replication fork [119,120].

Regarding responses to stress, the genes *ahpC* and *ahpF* play an important role in oxidative stress resistance in *M. catarrhalis. ahpC* encodes a thiol-dependent peroxiredoxin that catalyses the reduction of hydrogen peroxide (H₂O₂) [121,122], organic hydroperoxides such as tert-butyl hydroperoxide (t-BHP) and cumene hydroperoxide (CHP), and peroxynitrite, thereby protecting bacterial cells from oxidative damage [123,124]. *ahpF* encodes for a flavoprotein with oxidoreductase activity that regenerates the reduced form of *AhpC*, ensuring the continuation of its antioxidant function [122,125]. Although these genes are not unique to *M. catarrhalis* and are widely distributed among aerobic and facultative anaerobic bacteria, their presence and potential regulation under respiratory tract conditions where oxidative stress is a common immune defence mechanism suggest that they may contribute to the bacterium’s ability to survive within the host. In *Bacillus subtilis*, for example, *AhpC* is expressed as a general stress protein induced by adverse environmental conditions, including heat shock, osmotic stress and the stationary phase [126], indicating a broader evolutionary role in bacterial stress resilience.

Additionally, several genes associated with the formation of type IV pili were identified, including *pilA*, *pilB*, *pilC*, *pilF*, *pilO*, *pilP*, *pilQ*, *pilV* and *pilW*. These structures are known to play important roles in adhesion, twitching motility and horizontal gene transfer in many Gram-negative bacteria. PilA encodes the major subunit of the pilus filament [96], PilQ serves as the outer membrane secretin [96], and *PilT* is an ATPase involved in pilus retraction [127]. Although type IV pili genes are broadly conserved across many bacterial species and not restricted to the SR lineage of *M. catarrhalis*, their expression is regulated by iron availability via the Fur regulator, and their role in adherence and biofilm formation has been linked to colonization of the human respiratory tract. In contrast, genes such as *murA*, *murB*, *murC*, *murE*, *murF* and *murG*, which are involved in lipid II biosynthesis and form part of the conserved *dcw* (division and cell wall) cluster [128], are ubiquitous among bacteria and essential for the synthesis of peptidoglycan precursors [129]. While these genes are crucial for bacterial survival and cell division, their presence in *M. catarrhalis* is not explicitly associated with virulence or host adaptation and should be interpreted within the broader context of conserved bacterial physiology [126] (Table S2).

Taken together, these results highlight the presence of both conserved genes involved in fundamental cellular processes and others potentially linked to host adaptation in *M. catarrhalis*. While many of the identified genes, such as those related to DNA replication and repair, oxidative stress responses and peptidoglycan biosynthesis, are widely conserved across bacterial taxa, the presence of genes associated with adhesion and biofilm formation, such as those encoding components of the type IV pili, may contribute to colonization and persistence in the human respiratory tract. These features could facilitate the establishment of chronic infections, although experimental evidence is needed to confirm their specific roles in *M. catarrhalis* pathogenesis. Among the proteins encoded by these genes, PilQ and CopB are promising candidates for therapeutic targeting. PilQ, due to its surface localization and role in type IV pilus biogenesis, may serve as a target for interfering with bacterial adhesion and host colonization [130]. CopB, another surface-exposed outer membrane protein involved in nutrient acquisition, particularly iron, has been associated with host interaction and immune recognition [131], suggesting its potential as a therapeutic target.

Among the phylogroups A and B of *M. catarrhalis*, the genes *dppB*, *dppC* and *dppD* were identified as part of the Dpp (dipeptide permease) system [129], a transporter belonging to theATP-binding cassette (ABC) superfamily, responsible for the uptake of dipeptides and some tripeptides into the cell [130,132]. In *Helicobacter pylori*, the Dpp transporter consists of five proteins: DppA, DppB, DppC, DppD and DppF [131,133]. Within this system, DppB and DppC are membrane proteins that form the permease channel for the substrate, while DppD and DppF are cytoplasmic proteins responsible for ATP hydrolysis, driving the import of peptides into the cell [134]. Additionally, genes *moaA*, *moaB*, *moaC*, *moaD*, *moaE*, *modA* and *modC*, associated with molybdate metabolism, were identified. Molybdate is an essential cofactor for various bacterial enzymes. In bacteria, nitrate reductases depend on molybdenum cofactors to catalyse the reduction of nitrate to nitrite [135], a crucial process in anaerobic respiration and nitrogen assimilation [136]. The moaABCDE operon in *E. coli* plays a fundamental role in the biosynthesis of molybdopterin (MPT), a precursor molecule required for the activation of molybdenum-dependent enzymes [137]. In this process, MoaA, MoaB and MoaC catalyse the synthesis of precursor Z, while MPT synthase, formed by MoaD and MoaE, adds sulphur groups to form MPT [137].

These results highlight the presence of key metabolic systems exclusively in phylogroups A and B of *M. catarrhalis*, suggesting a differential adaptive capacity compared to phylogroup C. The identification of the Dpp transport system (DppB, DppC and DppD) implies a significant metabolic advantage for peptide and nutrient acquisition [131,133], which could be associated with increased efficiency in nutrient-rich environments within the host, such as the respiratory mucosa. Likewise, the exclusive presence of the *moa* operon in these phylogroups, involved in molybdate metabolism, suggests that they may exploit molybdenum-dependent enzymes for essential processes such as anaerobic respiration or nitrogen assimilation [135]. These differences may explain variations in colonization capacity, persistence and even virulence among phylogroups.

Additionally, the modA and modC genes encode molybdate transport proteins that facilitate the uptake of this essential metal into the cell. Between phylogroups A and C, the genes *pstA*, *pstB*, *pstC* and *pstS* were identified, encoding the components of the phosphate-specific transport (Pst) system [138]: PstS, a periplasmic phosphate-binding protein [139]; PstA and PstC, transmembrane proteins forming the transport channel; and PstB, a cytoplasmic protein with a nucleotide-binding domain essential for ATP hydrolysis and active phosphate transport [140]. On the other hand, between phylogroups B and C, the *ybaK* gene was identified, encoding an aminoacyl-tRNA deacylase involved in correcting errors in amino acid loading onto tRNAs [141]. In *H. influenzae*, YbaK has been characterized as a moderately specific aa-tRNA deacylase, capable of hydrolysing mischarged Cys-tRNA^Cys^, as well as other aa-tRNAs such as Gly-tRNA^Gly^, Ala-tRNA^Ala^, Ser-tRNA^Ser^, Pro-tRNA^Pro^ and Met-tRNA^Met^ [141]. Its activity is essential for translation homeostasis and the prevention of errors in protein synthesis (Table S2).

The different transport and metabolic systems identified among phylogroups suggest functional adaptations that may influence the pathogenic potential of *M. catarrhalis*. The exclusive presence of *modA* and *modC* in phylogroups A and B implies a greater reliance on molybdate-dependent enzymes, which could enhance survival in oxygen-limited environments, such as biofilms or inflamed tissues [142]. The Pst system in phylogroups A and C suggests alternative strategies for phosphate acquisition, a key element in bacterial virulence and stress adaptation [140]. Additionally, the presence of *ybaK* in phylogroups B and C, involved in maintaining translational fidelity, may contribute to protein homeostasis under stress conditions [141], potentially enhancing bacterial persistence. These metabolic differences could impact colonization efficiency, immune evasion and antibiotic resistance.

The phylogroups also exhibited specific genes. In phylogroup A, the *pgpB* gene was identified, which encodes a phosphatidylglycerophosphate phosphatase involved in phospholipid metabolism. In *E. coli*, the PgpB enzyme catalyses the conversion of phosphatidylglycerophosphate to phosphatidylglycerol, a crucial step in the biosynthesis of anionic phospholipids [143]. In phylogroup B, the *repB* gene was identified, which is involved in the regulation of extracellular enzyme and siderophore production in *Pseudomonas viridiflava* [144]. The *repB* locus encodes a response regulator homologous to *gacA*, a key component of two-component regulatory systems in *Pseudomonas syringae* and *Pseudomonas fluorescens* [145]. These systems play a fundamental role in regulating the production of virulence factors, such as pectate lyases, proteases and alginate, as well as being involved in iron acquisition through siderophores. In phylogroup C, the *oppA*, *oppB*, *oppC* and *oppF* genes were identified, encoding components of the oligopeptide permease system, an ABC transporter responsible for peptide uptake [27]. In *M. catarrhalis*, this system is crucial for the acquisition of arginine, an essential amino acid for bacterial survival and adaptation [146]. Additionally, this phylogroup also contained the *argC* and *argJ* genes, which encode enzymes involved in arginine biosynthesis. In *E. coli*, the *N*-acetylornithine transaminase (ArgD) fulfils transamination reactions in both the arginine biosynthetic pathway and the DAP pathway for lysine [147]. In *Corynebacterium glutamicum*, two distinct enzymes (ArgD and DapC) catalyse the reactions in the two pathways, respectively [148,149] (Table S2).

### Phylogenetic and genomic distinctions of phylogroup C: A potential new taxonomic entity?

The functional analysis of the genes identified in each phylogroup revealed differences in their genomic composition. Phylogroups A and B share a greater number of genes and exhibit a close phylogenetic relationship, in contrast to phylogroup C, which is more distantly related (Fig. 1b), shares fewer genes with the other two phylogroups and presents a higher number of unique genes. Additionally, the total genomic content varies among them: phylogroup A contains 1,710 ± 52 genes, phylogroup B 1,737 ± 50 and phylogroup C 1,877 ± 18. These differences in genetic composition, along with the low number of shared genes, may indicate an evolutionary divergence process specific to phylogroup C [20,22].

To understand the genomic relationship among the phylogroups (Fig. 1c), an ANI analysis based on ANIm was performed to assess the percentage of identity between genomes. The results showed that the similarity between phylogroups A and B was greater than 95%, indicating a high degree of genetic relationship between them. However, the comparison of phylogroup C with phylogroups A and B showed values below 95%, suggesting greater genomic divergence. Based on these results, a dDDH (Table 1) analysis was conducted for the genomes of phylogroup C (GCF_001656295.1, GCF_001656335.1, GCF_001656355.1 and GCF_001656375.1), which showed the highest similarity to *Moraxella canis*, with a coincidence index (C.I. d0, in %) ranging from 65.8–75.7%. In second place, these same genomes showed similarity to *M. catarrhalis*, with dDDH values ranging from 53.4–61.0 %, indicating a lower genomic relationship with this species. In both cases, a G+C content difference greater than 1% was observed, reinforcing the hypothesis that phylogroup C exhibits significant taxonomic divergence. To compare the genomic relationship with the other phylogroups, a representative strain of phylogroup A (GCF_000193045.1) and one from phylogroup B (GCF_001656415.1) were included in the analysis. The results showed that the phylogroup A strain had dDDH values between 95.0 and 97.9% with *M. catarrhalis*, confirming its classification within this species. In the case of phylogroup B, dDDH values ranged between 81.6 and 88.4 %, and the G+C content difference between phylogroups A and B was only 0.13%, indicating a closer phylogenetic relationship between them, despite some genomic variability within *M. catarrhalis*. These results suggest that while phylogroups A and B represent variations within *M. catarrhalis*, phylogroup C exhibits greater genomic divergence, potentially requiring reclassification within the *Moraxella* genus [19].

**Table 1.** dDDH values of phylogroup C. The table presents dDDH values obtained using three Genome blast Distance Phylogeny formulas: d0 (HSP length/total genome length), d4 (sum of identities in HSPs/HSP length) and d6 (sum of identities in HSPs/total genome length). The confidence intervals (C.I.) for each formula provide an estimate of variability in similarity values

Identifier	Subject strain	C.I. (d0, in %)	C.I. (d4, in %)	C.I. (d6, in %)	G+C content difference (in %)
GCF 001656295.1	*M. canis*	65.8–73.4	44.6–49.7	62.8–69.4	1.46
	*M. catarrhalis*	53.9–61.0	34.4–39.4	49.5–55.7	2.07
GCF 001656335.1	*M. canis*	68.1–75.7	45.0–50.1	64.8–71.5	1.39
	*M. catarrhalis*	53.6–60.7	34.0–39.0	49.1–55.3	2.14
GCF 001656355.1	*M. canis*	68.1–75.7	44.7–49.8	64.7–71.3	1.43
	*M. catarrhalis*	53.4–60.4	33.8–38.8	48.9–55.0	2.09
GCF 001656375.1	*M. canis*	68.1–75.7	45.0–50.2	64.8–71.5	1.4
	*M. catarrhalis*	53.5–60.6	33.9–38.9	49.0–55.2	2.13
GCF 000193045.1*	*M. catarrhalis*	95.0–97.9	90.2–93.9	96.4–98.4	0.13
	*M. canis*	37.7–44.5	24.4–29.2	33.5–39.5	3.66
GCF 001656415.1*	*M. catarrhalis*	81.6–88.4	62.4–68.1	81.2–87.2	0.13
	*M. canis*	38.2–45.0	24.7–29.6	34.0–40.0	3.66

*Correspond to the representative strain of phylogroup A (GCF_000193045.1) and one from phylogroup B (GCF_001656415.1).

**Fig. 2.**
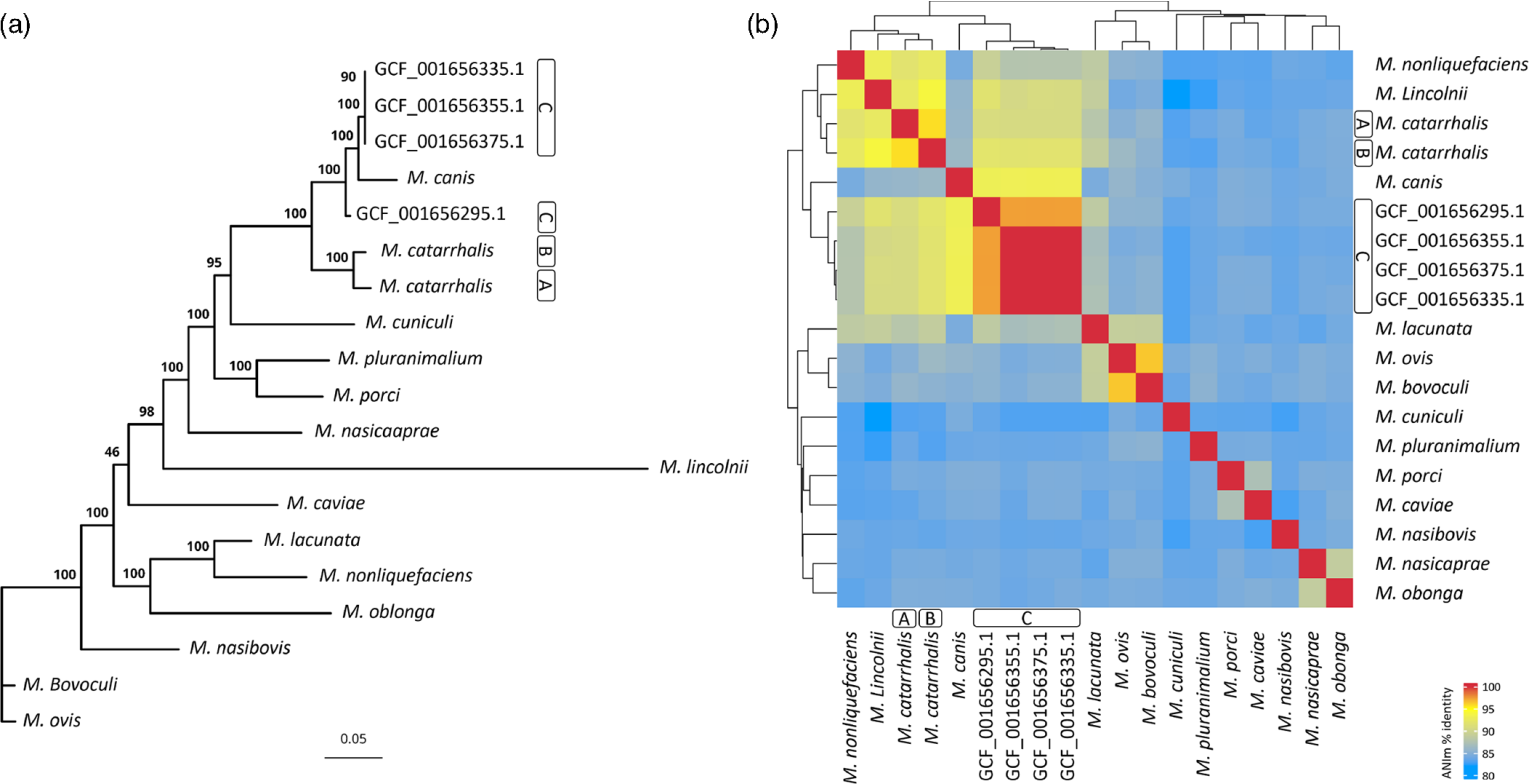
Phylogenetic and genomic differences of phylogroup C. (**a**) Maximum likelihood phylogenetic tree including various *Moraxella* species, highlighting the distinct positioning of phylogroup C. (**b**) Heatmap of ANI values calculated using Pyani, displaying the genomic similarity among the same *Moraxella* species analysed in the phylogenetic tree.

The phylogenetic analysis conducted with the species obtained from the dDDH results revealed that the genomes of phylogroup C exhibit a distinct evolutionary relationship within the *Moraxella* genus. The resulting phylogenetic tree showed a common node from which two main branches emerged (Fig. 2a), one grouping the representative strains of phylogroups A and B, confirming their close relationship, and another that subdivided into two clades. The first clade contains exclusively the GCF_001656295.1 strain, while the second splits into two sub-branches, one containing *M. canis* and the other comprising the genomes GCF_001656335.1, GCF_001656355.1 and GCF_001656375.1. These results indicate that while phylogroup C shows greater phylogenetic proximity to *M. canis* than to *M. catarrhalis*, there remains significant divergence within this group, suggesting that it may represent a distinct taxonomic entity within the *Moraxella* genus [19]. To complement this analysis, the ANI (Fig. 2b) was calculated among the same species included in the phylogenetic analysis. The results showed that the genomes of phylogroup C had 93% nucleotide identity with *M. canis*, while their identities with representatives of phylogroup A and phylogroup B were 91% and 92 %, respectively. The genomes of phylogroup C were classified as *Moraxella catarrhalis_C* by GTDB-Tk, a designation that indicates candidate species status within the GTDB framework. This classification suggests that while the genomes share sufficient similarity with the reference species to warrant a preliminary classification, unresolved differences still prevent a definitive taxonomic assignment [150]. The distinct genomic characteristics of phylogroup C, including its lower gene content overlap with phylogroups A and B, its higher proportion of unique genes and its greater genomic divergence as revealed by ANI and dDDH analyses, suggest that it may represent a separate taxonomic entity within the *Moraxella* genus. The ANI values below 95% when compared to phylogroups A and B, along with dDDH indices closer to *M. canis* than to *M. catarrhalis*, indicate that phylogroup C does not align with the genomic parameters of *M. catarrhalis*. Additionally, the G+C content difference exceeding 1% reinforces the hypothesis that it may belong to a different *Moraxella* species.

To determine whether phylogroup C constitutes a novel species or a distinct lineage within an existing *Moraxella* species, further studies are required. Phenotypic characterization is essential to assess whether it exhibits distinct metabolic or structural traits. Comparative functional genomics could clarify whether its unique genes provide specific ecological adaptations. Additionally, experimental validation of gene expression and function may help define its taxonomic placement. Given its genomic divergence, phylogroup C may represent more than just intraspecific variation and could be classified as a candidate species within *Moraxella*. However, formal classification would require meeting key taxonomic thresholds, such as dDDH values above 70% and ANI values exceeding 95% with a recognized species.

Based on the results suggesting that the members of phylogroup C may represent a species different from *M. catarrhalis*, subsequent analyses were conducted, excluding this group. Consequently, the pangenome and the corresponding phylogeny were reconstructed considering only the data from phylogroups A and B.

### Evaluation of genomic and epidemiological patterns

After reconstructing the pangenome using the 341 genomes belonging to phylogroups A and B, a total of 3,219 genes were identified and classified based on their frequency in the analysed genomes. Among these, 1,435 genes were identified as core genes, present in 100% of the genomes. Additionally, 30 soft-core genes (95%≤strains<100 %), 474 shell genes (15%≤strains<95 %) and 1,280 cloud genes (<15 % of the strains) were identified. The distribution of accessory genes (shell and cloud) is detailed in Figure S2. To assess the pangenome expansion dynamics, Heap’s Law was applied, yielding a *γ* value of 0.093. This represents a decrease compared to the previously obtained value when phylogroup C was included (*γ*=0.126), indicating a higher gene acquisition rate in the pangenome. The reduction in *γ* after removing phylogroup C suggests that this group made a significant contribution to the genetic variability of the species. These results support the hypothesis that phylogroup C could represent a distinct species, whose inclusion increased the gene acquisition rate and, consequently, the openness of the *M. catarrhalis* pangenome.

**Fig. 3.**
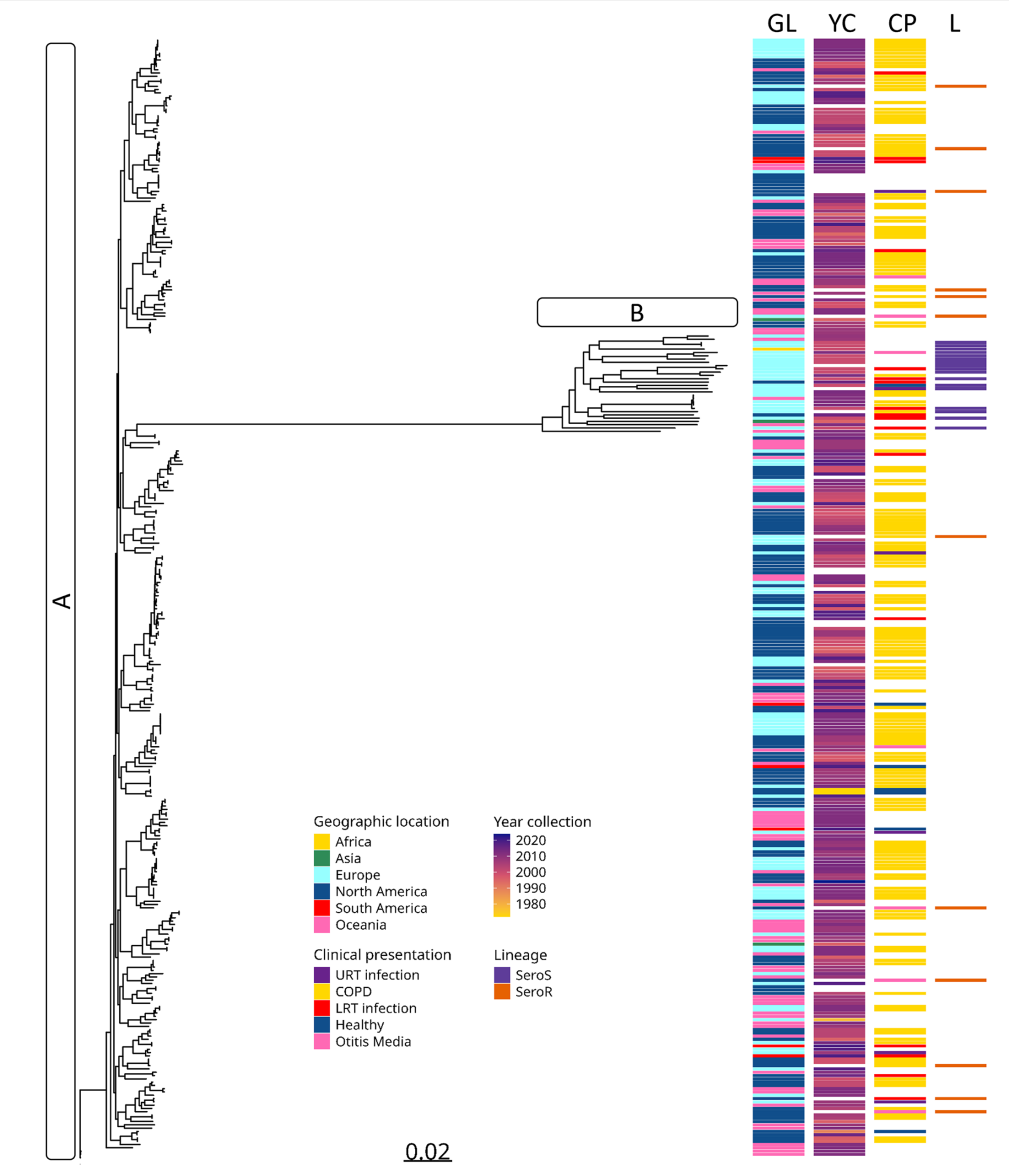
Genomic diversity and epidemiological factors of *M. catarrhalis*. Maximum likelihood phylogenetic tree of *M. catarrhalis*, considering only phylogroups A and B. The metadata includes geographic location (GL), year of collection (YC), clinical presentation (CP) and lineage classification (**l**). The letters A and B correspond to the classification of phylogroups: A represents the SR lineage and B the SS lineage.

To further understand the relationship between genomic diversity and epidemiological factors, phylogenetic analysis of phylogroups A and B was correlated with available epidemiological data (Fig. 3), including geographic location, year of isolation and host condition at the time of sampling. However, no clear relationship was observed between the phylogenetic structure and these variables, suggesting that the genomic diversity of *M. catarrhalis* is not strongly influenced by geographical or temporal factors. In contrast, a clear correlation was found with the previously reported SR and SS lineages [19,21]. Isolates belonging to phylogroup A were predominantly associated with the SR lineage, while those in phylogroup B corresponded mainly to the SS lineage. This classification aligns with previous studies that have reported differences in complement system susceptibility and virulence between these lineages.

### Functional characterization of the core genome of *M. catarrhalis*

**Fig. 4.**
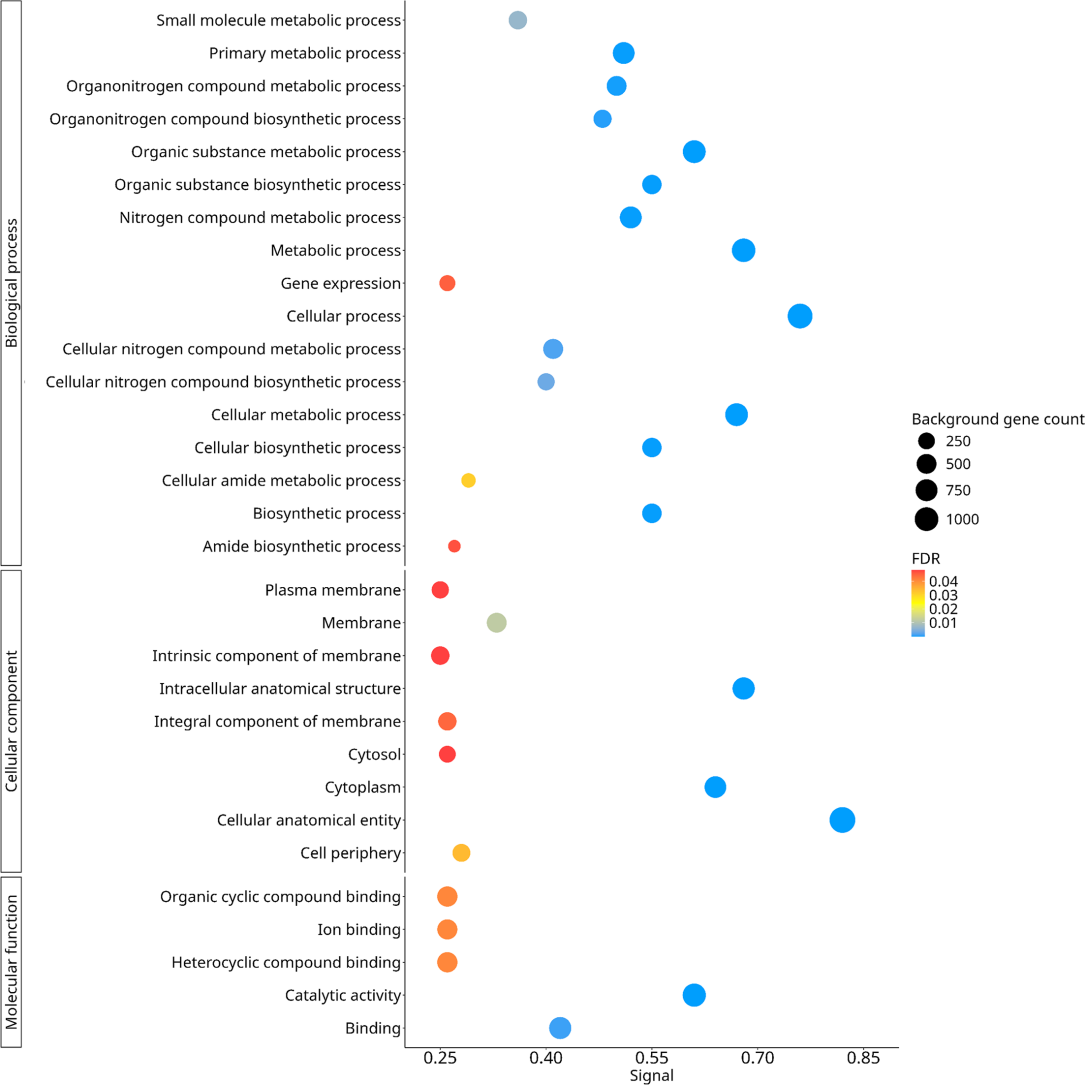
Functional analysis of the core genome of *M. catarrhalis*. GO enrichment analysis of the core genome genes. GO terms are classified into biological process, cellular component and molecular function. Each term is represented by a circle, where the size indicates the number of associated genes, and the colour reflects the FDR.

The analysis of the core genes of *M. catarrhalis* using GO (Fig. 4) revealed that most of these proteins are involved in essential functions for bacterial survival and adaptation, encompassing key structural, functional and metabolic aspects.

In the cellular components category, proteins related to cellular structure and organization were identified, including cellular anatomical entity (1,042 proteins) and intracellular anatomical structure (634 proteins). Likewise, proteins associated with the plasma membrane and its components were identified, including membrane proteins (374 proteins), intrinsic membrane components (276 proteins), integral membrane components (274 proteins), proteins at the cell periphery (253 proteins) and plasma membrane proteins (219 proteins). These membrane proteins play key roles in epithelial surface adhesion, cell recognition and evasion of the immune system [151]. Some of these membrane proteins have been previously associated with functions such as epithelial surface adhesion, cell recognition and immune evasion. Their presence in *M. catarrhalis* suggests a potential role in host interaction and persistence in the respiratory tract. Moreover, the lateral dynamics of these membrane components regulate protein–protein and lipid–protein interactions [150,152,154], influencing cell signalling mechanisms and environmental adaptation. The high representation of proteins associated with the membrane highlights the importance of these processes in the biology of *M. catarrhalis*, particularly in its ability to colonize the respiratory tract and evade host immune responses [155].

In addition, proteins related to cellular structure, organization and the membrane were also identified, with high representation in the cytoplasm (569 proteins) and cytosol (201 proteins). Since the cytoplasm is the primary site for metabolic activity, protein synthesis and gene regulation [156], the abundance of these proteins suggests a key role in the adaptive response and cellular viability of *M. catarrhalis*.

Some of these cytoplasmic proteins, traditionally considered intracellular, can be exported into the extracellular environment through different mechanisms, acquiring additional functions known as ‘moonlighting’ proteins [157,158]. In *M. catarrhalis*, these proteins can be released directly or via outer membrane vesicles (OMVs), which serve as transport systems for virulence factors and play a crucial role in immune evasion [159]. OMVs contain numerous surface and periplasmic proteins, such as UspA1, UspA2 and MID (*Moraxella* IgD-binding protein), many of which contribute to host immune modulation by interfering with complement activation and suppressing pro-inflammatory responses [159]. For instance, UspA1 has been shown to bind to carcinoembryonic antigen-related cell adhesion molecule 1, thereby reducing the inflammatory response triggered by Toll-like receptor 2 [158,159]. Furthermore, proteomic analyses have identified OMV-associated proteins, including CopB and OMP E, that induce immune responses, making them promising candidates for vaccine development [160]. Given their role in virulence and persistence, some of these moonlighting proteins, such as metabolic enzymes like enolase and glyceraldehyde-3-phosphate dehydrogenase, may act as key targets for novel therapeutic strategies against *M. catarrhalis* infections [161].

In the molecular function category, proteins related to catalytic activity (709 proteins) and molecular binding processes (572 proteins) were identified. Among these, proteins involved in heterocyclic compound binding (407 proteins), organic cyclic compound binding (407 proteins) and ion binding (394 proteins) were predominant. These binding processes play a crucial role in enzymatic activity, molecular interactions and bacterial adaptation [162].

Proteins involved in catalytic activity are crucial for accelerating biochemical reactions within the cell. Enzymes act as biological catalysts, increasing reaction rates by several orders of magnitude. Without enzymatic catalysis, most cellular reactions would proceed too slowly to sustain life under physiological conditions [163]. In *M. catarrhalis*, one such catalytic enzyme is the glucosyltransferase Lgt3, which plays a crucial role in the biosynthesis of LOS [164]. LOS are glycolipid surface molecules that contribute to bacterial colonization and virulence [165]. Lgt3 contains two distinct glycosyltransferase domains (A1 and A2), each possessing a conserved DXD motif [166], which is critical for catalytic activity. The N-terminal A1 domain is responsible for adding the first *β*-(1-3) glucose (Glc) residue to the inner core of the LOS molecule, while the C-terminal A2 domain sequentially incorporates *β*-(1-4) Glc and *β*-(1-6) Glc, highlighting its bifunctional nature [167]. This enzymatic activity is essential for the structural integrity of LOS, which in turn influences bacterial immune evasion and host interactions. Nevertheless, whether other enzymes with similar binding functions directly contribute to host adaptation remains to be demonstrated through functional and comparative studies.

In the biological processes category, proteins related to cellular metabolism (685 proteins), organic compound metabolism (675 proteins) and nitrogen compound metabolism (566 proteins) were identified. Additionally, proteins associated with cellular biosynthesis (381 proteins), small molecule metabolism (297 proteins) and gene expression (176 proteins) were present.

Metabolism-associated proteins in *M. catarrhalis* include those involved in the conversion of large organic compounds, such as carbohydrates processed through catabolic pathways, into smaller products like organic acids and carbon dioxide [168]. However, unlike other bacterial pathogens, *M. catarrhalis* lacks a complete glycolytic pathway and instead relies on amino acid and organic acid metabolism as alternative energy sources [169]. Additionally, it possesses a functional glyoxylate cycle, allowing it to utilize acetate and simple carbon compounds for energy production, facilitating its adaptation to nutrient-limited environments [169]. These findings suggest that *M. catarrhalis* has evolved metabolic strategies to support survival under restrictive conditions; nevertheless, confirming the biological relevance of these adaptations requires further experimental validation, particularly through metabolic flux analyses and gene knockout approaches.

Regarding nitrogen metabolism, *M. catarrhalis* has developed strategies that enable its persistence within the host. It expresses pathways for ammonia assimilation, including glutamate dehydrogenase and glutamate synthase-glutamine synthetase systems. However, it lacks key regulatory elements such as RpoN, PII, NtrB and NtrC, which are common in other bacteria [169,170]. Instead, *M. catarrhalis* predominantly degrades alanine, arginine, glycine and histidine as nitrogen sources, reinforcing its metabolic flexibility and adaptive capacity to the conditions of the respiratory tract [170].

The high representation of genes involved in cellular metabolism and biosynthesis suggests that *M. catarrhalis* has developed efficient strategies to adapt to fluctuations in nutrient availability. Its dependence on alternative energy sources and the absence of carbohydrate catabolism pathways indicate an evolutionary adaptation that enhances its survival in highly competitive niches with limited resources, such as the respiratory tract.

### *β*-Lactamase-mediated resistance in *M. catarrhalis*

The antibiotic resistance analysis revealed that phylogroup A harboured resistance genes in 51.45 % of the strains, with 47.59% carrying the *bro-1* gene and 2.89% carrying the *bro-2 gene*. In phylogroup B, the prevalence of resistance genes was significantly higher, reaching 80.00%, with 70.00% of strains harbouring *bro-1* and 6.67% carrying *bro-2*.

The *bro-1* and *bro-2* genes encode BRO *β*-lactamases, the primary mediators of *M. catarrhalis* resistance to *β*-lactam antibiotics, such as amoxicillin and penicillin. While both enzymes exhibit similar substrate profiles and inhibition patterns, BRO-1 confers a higher level of resistance than BRO-2, likely due to its higher expression levels in *bro-1*-positive strains [171]. The difference in the expression of these *β*-lactamases appears to be associated with promoter sequence variations, where a 21-bp deletion in the *bro-2* promoter has been identified, potentially affecting its transcription [171].

Previous studies have reported that over 90% of clinical isolates of *M. catarrhalis* worldwide produce *β*-lactamases, with *bro-1* being more prevalent than *bro-2* [172]. Moreover, the subcellular localization of BRO *β*-lactamases suggests that they function as outer membrane-associated lipoproteins, which may enhance bacterial resistance to *β*-lactam antibiotics and contribute to persistence within the host [173].

The predominance of the *bro-1* gene in both phylogroups aligns with previous findings, highlighting its greater impact on *β*-lactam resistance compared to the *bro-2 gene*. Additionally, variability in *bro-1* and *bro-2* expression suggests potential differences in genetic regulation of resistance between these phylogroups, which may be influenced by selective pressures in distinct clinical environments. The association of BRO *β*-lactamases with the outer membrane may further contribute to the stability of resistance, enhancing immune evasion and bacterial persistence in the respiratory tract.

### Identification of virulence-associated proteins in *M. catarrhalis*

**Fig. 5.**
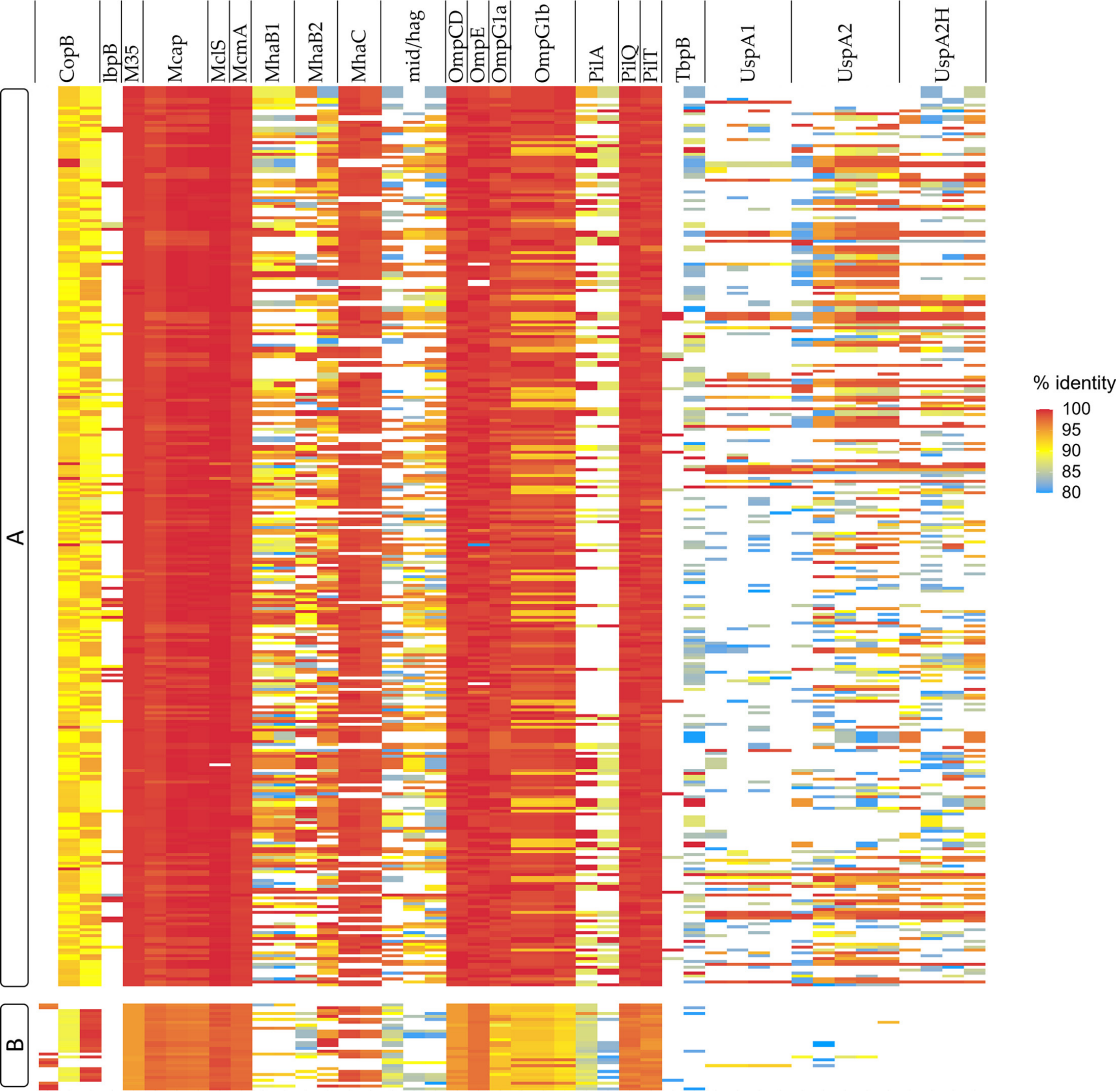
Virulence factor profiling in *M. catarrhalis*. Heatmap based on conserved virulence factors according to blast results, with identity and coverage percentages above 80%. Columns represent specific proteins associated with virulence, while rows correspond to different *M. catarrhalis* genomes. The letters A and B indicate the classification of the phylogroups.

The identification of virulence-associated proteins in *M. catarrhalis* revealed that several virulence factors are conserved across the analysed genomes (Fig. 5). Among them, M35, McaP, McmA, OmpCD, OmpG1b, PilQ and PilT play essential roles in host adhesion, nutrient acquisition and the formation of specialized structures for environmental interaction (Table S3).

Among the proteins involved in host adhesion, McaP, OmpCD and OmpG1b were identified. McaP is an outer membrane adhesin that facilitates the attachment of *M. catarrhalis* to human epithelial cells, particularly pulmonary cells. Its N-terminal passenger domain is responsible for interacting with the cell surface, enhancing colonization stability [85]. OmpCD plays a crucial role in adhesion, as its presence significantly increases the ability of *M. catarrhalis* to bind to respiratory epithelial cells [81]. Additionally, it contributes to the structural stability of the outer membrane, which may influence bacterial resistance to adverse conditions [81]. OmpG1b, although not yet fully characterized, has been associated with membrane permeability and host interaction, suggesting a potential role in adhesion and bacterial structural stability [174].

In nutrient acquisition, the presence of outer membrane porins is essential for the uptake of molecules necessary for metabolism and survival within the host. In this process, M35 stands out as a key player. M35 is an outer membrane porin that plays a fundamental role in regulating permeability, allowing the passage of essential nutrients [80]. Its deletion negatively impacts the bacterium’s ability to survive in resource-limited environments, highlighting its importance in the metabolic adaptation of *M. catarrhalis* [80].

Finally, in the formation of specialized structures, the proteins PilQ and PilT are crucial for the biogenesis and function of type IV pili, structures essential for bacterial motility, adhesion and genetic transformation. PilQ forms a channel in the outer membrane through which type IV pili emerge [175]. These structures enable the bacterium to adhere to cellular surfaces and form microcolonies, facilitating its persistence within the host. PilT is an ATPase responsible for the retraction of type IV pili. This process is fundamental for bacterial motility and the uptake of environmental DNA, contributing to genetic variability and bacterial adaptation to new conditions [176].

Together, these proteins play a crucial role in the biology of *M. catarrhalis*, enabling it to colonize the host, acquire essential nutrients and develop specialized structures that promote its persistence and environmental adaptation. The interaction of adhesins such as McaP, OmpCD and OmpG1b with respiratory tract epithelial cells facilitates bacterial establishment in the host, while the presence of porins like M35 enables the uptake of key metabolic molecules. Additionally, the involvement of PilQ and PilT in the biogenesis and retraction of type IV pili highlights the importance of these structures in adhesion, motility and potential genetic transfer, contributing to the bacterium’s evolutionary plasticity. The conservation of these factors across the analysed genomes suggests that they play critical roles in *M. catarrhalis* survival in hostile environments, underscoring their significance in pathogenesis and their ability to adapt to various ecological niches.

It is essential to acknowledge that the majority of genomes analysed in this study were derived from clinical isolates, primarily obtained from patients with respiratory conditions, such as COPD. This sampling bias may influence the observed pangenome size, virulence factor distribution and overall genetic diversity, as strains associated with disease may differ from those carried asymptomatically in healthy individuals. Future studies incorporating a broader range of commensal and environmental isolates will be crucial to fully capture the genetic diversity and ecological dynamics of *M. catarrhalis*.

## Conclusion

The phylogenomic analysis of *M. catarrhalis* revealed three distinct phylogroups exhibiting significant genetic differences. However, ANI and dDDH results indicated that one of them exhibited considerable divergence from the other two, with values below the genomic identity thresholds established for *M. catarrhalis*. This divergence suggests that this phylogroup could represent a new species within the *Moraxella* genus and was, therefore, excluded from further analyses. After its removal, the pangenome reconstruction showed a reduction in the gene acquisition rate, indicating that this divergent phylogroup was a key factor in the initially observed genetic variability.

The analysis of the two remaining phylogroups allowed for a detailed characterization of their functional differences and their association with the previously described seroresistant (phylogroup A) and serosensitive (phylogroup B) lineages. One of the key differences between them was the prevalence of antibiotic resistance genes. Phylogroup B exhibited a higher prevalence of resistance genes, with 80.00% of strains carrying at least one resistance determinant, including 70.00% harbouring *bro-1* and 6.67% carrying *bro-2*. In contrast, phylogroup A had a lower proportion of resistant strains, with 51.45% carrying resistance genes, including 47.59% with *bro-1* and 2.89% with *bro-2*. These findings suggest that phylogroup B may have a greater capacity to withstand *β*-lactam antibiotics, whereas phylogroup A may rely on alternative mechanisms for persistence and survival.

Both phylogroups shared key virulence factors, including outer membrane proteins such as UspA1 and UspA2, which play essential roles in immune evasion and adhesion to epithelial cells. However, they differed in transport and metabolic systems. Phylogroup A contained genes for the DppB-DppC-DppD dipeptide transport system. In contrast, phylogroup B exhibited genes for the molybdate metabolism pathway, including *moaA*, *moaB*, *moaC*, *moaD*, *moaE*, *modA* and *modC*. Additionally, AhpC, a key protein for oxidative stress resistance, was identified in both phylogroups.

These differences in genetic composition suggest that phylogroup B may have a greater ability to resist antibiotics, while phylogroup A exhibits adaptations related to alternative nutrient acquisition and stress response. Despite these distinctions, both phylogroups share key virulence factors, highlighting common strategies for host colonization and persistence. Given these findings, it is crucial to identify molecular targets involved in these processes. Among them, PilQ is essential for the formation of type IV pili, playing a critical role in DNA uptake, twitching motility and interactions with host cells in *M. catarrhalis* [90]. This structural protein forms a multimeric channel in the outer membrane, facilitating pilus extrusion and retraction mechanisms involved in adhesion and colonization [90]. Similarly, CopB is an immunogenic outer membrane protein whose expression is strongly induced under iron-limited conditions. It contributes to iron acquisition through interaction with transferrin and promotes bacterial survival in iron-restricted environments [4]. Its surface exposure and antigenic properties have led to its proposal as a vaccine candidate [81]. Together, these proteins exemplify the adaptive strategies of *M. catarrhalis* that enable it to persist in the host environment and highlight the potential for targeting conserved components involved in virulence and survival.

## Supplementary material

10.1099/mgen.0.001488 Uncited Fig. S1.

10.1099/mgen.0.001488 Uncited Table S1.

10.1099/mgen.0.001488 Uncited Table S2.

10.1099/mgen.0.001488 Uncited Table S3.

## Abbreviations

ABC: ATP-binding cassette
ANI: average nucleotide identity
C.I.: confidence intervals
COPD: chronic obstructive pulmonary disease
dDDH: digital DNA–DNA hybridization
DDH: DNA–DNA hybridization
FDR: false discovery rate
GO: Gene Ontology
LOS: lipooligosaccharide
MGEs: mobile genetic elements
MLST: multilocus sequence typing
MPT: molybdopterin
NCBI: National Center for Biotechnology Information
OMPs: outer membrane proteins
OMVs: outer membrane vesicles
Pst: phosphate-specific transport
RB: ribotype
SR: seroresistant
SRA: Sequence Read Archive
SS: serosensitive
UspA: ubiquitous surface protein A

## References

[1] Paykel JM (2002). Moraxella (branhamella) catarrhalis infections. Prim Care Update Ob Gyns.

[2] Spaniol V, Troller R, Schaller A, Aebi C (2011). Physiologic cold shock of moraxella catarrhalis affects the expression of genes involved in the iron acquisition, serum resistance and immune evasion. BMC Microbiol.

[3] Del Beccaro MA, Mendelman PM, Inglis AF, Richardson MA, Duncan NO (1992). Bacteriology of acute otitis media: a new perspective. J Pediatr.

[4] Karalus R, Campagnari A (2000). Moraxella catarrhalis: a review of an important human mucosal pathogen. Microbes Infect.

[5] Faden H, Bernstein J, Brodsky L, Stanievich J, Ogra PL (1992). Effect of prior antibiotic treatment on middle ear disease in children. Ann Otol Rhinol Laryngol.

[6] Ruohola A, Meurman O, Nikkari S, Skottman T, Salmi A (2006). Microbiology of acute otitis media in children with tympanostomy tubes: prevalences of bacteria and viruses. Clin Infect Dis.

[7] Murphy TF, Parameswaran GI (2009). Moraxella catarrhalis, a human respiratory tract pathogen. Clin Infect Dis.

[8] Wald ER (1998). Microbiology of acute and chronic sinusitis in children and adults. Am J Med Sci.

[9] Brook I, Foote PA, Hausfeld JN (2006). Frequency of recovery of pathogens causing acute maxillary sinusitis in adults before and after introduction of vaccination of children with the 7-valent pneumococcal vaccine. J Med Microbiol.

[10] Murphy TF, Brauer AL, Grant BJB, Sethi S (2005). Moraxella catarrhalis in chronic obstructive pulmonary disease: burden of disease and immune response. Am J Respir Crit Care Med.

[11] Doern GV (1986). *Branhamella catarrhalis*--an emerging human pathogen. Diagn Microbiol Infect Dis.

[12] Catlin BW (1990). *Branhamella catarrhalis*: an organism gaining respect as a pathogen. Clin Microbiol Rev.

[13] Daoud A, Abuekteish F, Masaadeh H (1996). Neonatal meningitis due to *Moraxella catarrhalis* and review of the literature. Ann Trop Paediatr.

[14] Verduin CM, Hol C, Fleer A, van Dijk H, van Belkum A (2002). *Moraxella catarrhalis*: from emerging to established pathogen. Clin Microbiol Rev.

[15] Patterson TF, Patterson JE, Masecar BL, Barden GE, Hierholzer WJ Jr (1988). A nosocomial outbreak of *Branhamella catarrhalis* confirmed by restriction endonuclease analysis. J Infect Dis.

[16] Richards SJ, Greening AP, Enright MC, Morgan MG, McKenzie H (1993). Outbreak of *Moraxella catarrhalis* in a respiratory unit. Thorax.

[17] Bootsma HJ, van der Heide HG, van de Pas S, Schouls LM, Mooi FR (2000). Analysis of *Moraxella catarrhalis* by DNA typing: evidence for a distinct subpopulation associated with virulence traits. J Infect Dis.

[18] Pingault NM, Lehmann D, Bowman J, Riley TV (2007). A comparison of molecular typing methods for *Moraxella catarrhalis*. J Appl Microbiol.

[19] Earl JP, de Vries SPW, Ahmed A, Powell E, Schultz MP (2016). Comparative genomic analyses of the *Moraxella catarrhalis* serosensitive and seroresistant lineages demonstrate their independent evolution. Genome Biol Evol.

[20] Meier PS, Troller R, Heiniger N, Grivea IN, Syrogiannopoulos GA (2005). *Moraxella catarrhalis* strains with reduced expression of the UspA outer membrane proteins belong to a distinct subpopulation. Vaccine.

[21] Wirth T, Morelli G, Kusecek B, van Belkum A, van der Schee C (2007). The rise and spread of a new pathogen: seroresistant Moraxella catarrhalis. Genome Res.

[22] Verhaegh SJC, Streefland A, Dewnarain JK, Farrell DJ, van Belkum A (2008). Age-related genotypic and phenotypic differences in *Moraxella catarrhalis* isolates from children and adults presenting with respiratory disease in 2001-2002. *Microbiology (Reading*).

[23] Shi W, Wen D, Chen C, Yuan L, Gao W (2018). β-Lactamase production and antibiotic susceptibility pattern of *Moraxella catarrhalis* isolates collected from two county hospitals in China. BMC Microbiol.

[24] Raveendran S, Kumar G, Sivanandan RN, Dias M (2020). *Moraxella catarrhalis*: a cause of concern with emerging resistance and presence of BRO beta-lactamase gene-report from a tertiary care hospital in South India. Int J Microbiol.

[25] Deshpande LM, Sader HS, Fritsche TR, Jones RN (2006). Contemporary prevalence of BRO β-lactamases in *Moraxella catarrhalis*: report from the SENTRY Antimicrobial Surveillance Program (North America, 1997 to 2004). J Clin Microbiol.

[26] Khan MA, Northwood JB, Levy F, Verhaegh SJC, Farrell DJ (2010). bro {beta}-lactamase and antibiotic resistances in a global cross-sectional study of *Moraxella catarrhalis* from children and adults. J Antimicrob Chemother.

[27] de Vries SPW, van Hijum SAFT, Schueler W, Riesbeck K, Hays JP (2010). Genome analysis of *Moraxella catarrhalis* strain BBH18, [corrected] a human respiratory tract pathogen. J Bacteriol.

[28] Spaniol V, Bernhard S, Aebi C (2015). *Moraxella catarrhalis* AcrAB-OprM efflux pump contributes to antimicrobial resistance and is enhanced during cold shock response. Antimicrob Agents Chemother.

[29] Jetter M, Spaniol V, Troller R, Aebi C (2010). Down-regulation of porin M35 in *Moraxella catarrhalis* by aminopenicillins and environmental factors and its potential contribution to the mechanism of resistance to aminopenicillins. J Antimicrob Chemother.

[30] Hall-Stoodley L, Hu FZ, Gieseke A, Nistico L, Nguyen D (2006). Direct detection of bacterial biofilms on the middle-ear mucosa of children with chronic otitis media. JAMA.

[31] Matejka KM, Bremer PJ, Tompkins GR, Brooks HJL (2012). Antibiotic susceptibility of *Moraxella catarrhalis* biofilms in a continuous flow model. Diagn Microbiol Infect Dis.

[32] Budhani RK, Struthers JK (1998). Interaction of Streptococcus pneumoniae and *Moraxella catarrhalis*: investigation of the indirect pathogenic role of beta-lactamase-producing moraxellae by use of a continuous-culture biofilm system. Antimicrob Agents Chemother.

[33] Høiby N, Bjarnsholt T, Givskov M, Molin S, Ciofu O (2010). Antibiotic resistance of bacterial biofilms. Int J Antimicrob Agents.

[34] Su Y-C, Singh B, Riesbeck K (2012). *Moraxella catarrhalis*: from interactions with the host immune system to vaccine development. Future Microbiol.

[35] Su Y-C, Hallström BM, Bernhard S, Singh B, Riesbeck K (2013). Impact of sequence diversity in the *Moraxella catarrhalis* UspA2/UspA2H head domain on vitronectin binding and antigenic variation. Microbes Infect.

[36] Hoiczyk E, Roggenkamp A, Reichenbecher M, Lupas A, Heesemann J (2000). Structure and sequence analysis of Yersinia YadA and Moraxella UspAs reveal a novel class of adhesins. EMBO J.

[37] Spaniol V, Heiniger N, Troller R, Aebi C (2008). Outer membrane protein UspA1 and lipooligosaccharide are involved in invasion of human epithelial cells by *Moraxella catarrhalis*. Microbes Infect.

[38] Brooks MJ, Sedillo JL, Wagner N, Wang W, Attia AS (2008). *Moraxella catarrhalis* binding to host cellular receptors is mediated by sequence-specific determinants not conserved among all UspA1 protein variants. Infect Immun.

[39] Hallström T, Nordström T, Tan TT, Manolov T, Lambris JD (2011). Immune evasion of *Moraxella catarrhalis* involves ubiquitous surface protein A-dependent C3d binding. J Immunol.

[40] N’Guessan PD, Vigelahn M, Bachmann S, Zabel S, Opitz B (2007). The UspA1 protein of *Moraxella catarrhalis* induces CEACAM-1-dependent apoptosis in alveolar epithelial cells. J Infect Dis.

[41] Meier PS, Troller R, Grivea IN, Syrogiannopoulos GA, Aebi C (2002). The outer membrane proteins UspA1 and UspA2 of *Moraxella catarrhalis* are highly conserved in nasopharyngeal isolates from young children. Vaccine.

[42] Lafontaine ER, Cope LD, Aebi C, Latimer JL, McCracken GH (2000). The UspA1 protein and a second type of UspA2 protein mediate adherence of *Moraxella catarrhalis* to human epithelial cells in vitro. J Bacteriol.

[43] Attia AS, Lafontaine ER, Latimer JL, Aebi C, Syrogiannopoulos GA (2005). The UspA2 protein of *Moraxella catarrhalis* is directly involved in the expression of serum resistance. Infect Immun.

[44] Pearson MM, Hansen EJ (2007). Identification of gene products involved in biofilm production by *Moraxella catarrhalis* ETSU-9 in vitro. Infect Immun.

[45] Nordström T, Blom AM, Tan TT, Forsgren A, Riesbeck K (2005). Ionic binding of C3 to the human pathogen *Moraxella catarrhalis* is a unique mechanism for combating innate immunity. J Immunol.

[46] Nordström T, Blom AM, Forsgren A, Riesbeck K (2004). The emerging pathogen *Moraxella catarrhalis* interacts with complement inhibitor c4b binding protein through ubiquitous surface proteins A1 and A2. J Immunol.

[47] Attia AS, Ram S, Rice PA, Hansen EJ (2006). Binding of vitronectin by the moraxella catarrhalis uspa2 protein interferes with late stages of the complement cascade. Infect Immun.

[48] National Center for Biotechnology Information Home - SRA. https://www.ncbi.nlm.nih.gov/sra/.

[49] Chen S, Zhou Y, Chen Y, Gu J (2018). fastp: an ultra-fast all-in-one FASTQ preprocessor. Bioinformatics.

[50] National Ceenter for Biotechnology Information Genome. https://www.ncbi.nlm.nih.gov/datasets/genome/.

[51] Chen S (2023). Ultrafast one-pass FASTQ data preprocessing, quality control, and deduplication using fastp. *Imeta*.

[52] Wood DE, Lu J, Langmead B (2019). Improved metagenomic analysis with Kraken 2. bioRxiv.

[53] Lu J, Rincon N, Wood DE, Breitwieser FP, Pockrandt C (2022). Metagenome analysis using the Kraken software suite. Nat Protoc.

[54] Prjibelski A, Antipov D, Meleshko D, Lapidus A, Korobeynikov Aus (2020). Using SPAdes DE novo assembler. CP in Bioinformatics.

[55] Chklovski A, Parks DH, Woodcroft BJ, Tyson GW (2023). CheckM2: a rapid, scalable and accurate tool for assessing microbial genome quality using machine learning. Nat Methods.

[56] Jolley KA, Bliss CM, Bennett JS, Bratcher HB, Brehony C (2012). Ribosomal multilocus sequence typing: universal characterization of bacteria from domain to strain. Microbiology.

[57] Jolley KA, Bray JE, Maiden MCJ (2018). Open-access bacterial population genomics: BIGSdb software, the PubMLST.org website and their applications. Wellcome Open Res.

[58] Seemann T (2022). Github.

[59] Davie JJ, Earl J, de Vries SPW, Ahmed A, Hu FZ (2011). Comparative analysis and supragenome modeling of twelve Moraxella catarrhalis clinical isolates. BMC Genomics.

[60] Schwengers O, Jelonek L, Dieckmann MA, Beyvers S, Blom J (2021). Bakta: rapid and standardized annotation of bacterial genomes via alignment-free sequence identification. Microb Genom.

[61] Tonkin-Hill G, MacAlasdair N, Ruis C, Weimann A, Horesh G (2020). Producing polished prokaryotic pangenomes with the Panaroo pipeline. Genome Biol.

[62] Katoh K, Standley DM (2013). MAFFT multiple sequence alignment software version 7: improvements in performance and usability. Mol Biol Evol.

[63] Stamatakis A (2014). RAxML version 8: a tool for phylogenetic analysis and post-analysis of large phylogenies. Bioinformatics.

[64] Flouri T, Izquierdo-Carrasco F, Darriba D, Aberer AJ, Nguyen L-T (2015). The phylogenetic likelihood library. Syst Biol.

[65] Kozlov AM, Aberer AJ, Stamatakis A (2015). ExaML version 3: a tool for phylogenomic analyses on supercomputers. Bioinformatics.

[66] Scholl C, Kobert K, Flouri T, Stamatakis A 2016 IEEE International Parallel and Distributed Processing Symposium Workshops (IPDPSW); Chicago, IL, USA, 2016.

[67] Balaban M, Moshiri N, Mai U, Jia X, Mirarab S (2019). TreeCluster: clustering biological sequences using phylogenetic trees. PLoS One.

[68] Alam S, Dobbie G, Koh YS, Riddle P, Ur Rehman S (2014). Research on particle swarm optimization based clustering: a systematic review of literature and techniques. Swarm Evol Comput.

[69] Zhang B, Ren H, Wang X, Han C, Jin Y (2024). Comparative genomics analysis to explore the biodiversity and mining novel target genes of *Listeria monocytogenes* strains from different regions. Front Microbiol.

[70] Tettelin H, Riley D, Cattuto C, Medini D (2008). Comparative genomics: the bacterial pan-genome. Curr Opin Microbiol.

[71] Heap_Law_for_Roary (2021). Github.

[72] Szklarczyk D, Kirsch R, Koutrouli M, Nastou K, Mehryary F (2023). The STRING database in 2023: protein-protein association networks and functional enrichment analyses for any sequenced genome of interest. Nucleic Acids Res.

[73] Benjamini Y, Hochberg Y (1995). Controlling the false discovery rate: a practical and powerful approach to multiple testing. J R Stat Soc Series B Stat Methodol.

[74] Feldgarden M, Brover V, Haft DH, Prasad AB, Slotta DJ (2019). Validating the AMRFinder tool and resistance gene database by using antimicrobial resistance genotype-phenotype correlations in a collection of isolates. Antimicrob Agents Chemother.

[75] Feldgarden M, Brover V, Gonzalez-Escalona N, Frye JG, Haendiges J (2021). AMRFinderPlus and the Reference Gene Catalog facilitate examination of the genomic links among antimicrobial resistance, stress response, and virulence. Sci Rep.

[76] Blakeway LV, Tan A, Peak IRA, Seib KL (2017). Virulence determinants of Moraxella catarrhalis: distribution and considerations for vaccine development. *Microbiology (Reading*).

[77] Chan C, Ng D, Schryvers AB (2021). The role of the *Moraxella catarrhalis* CopB protein in facilitating iron acquisition from human transferrin and lactoferrin. Front Microbiol.

[78] Morris DE, Osman KL, Cleary DW, Clarke SC (2022). The characterization of *Moraxella catarrhalis* carried in the general population. Microb Genom.

[79] Bonnah RA, Wong H, Loosmore SM, Schryvers AB (1999). Characterization of moraxella (branhamella) catarrhalis lbpb, lbpa, and lactoferrin receptor orf3 isogenic mutants. Infect Immun.

[80] Easton DM, Maier E, Benz R, Foxwell AR, Cripps AW (2008). *Moraxella catarrhalis* M35 is a general porin that is important for growth under nutrient-limiting conditions and in the nasopharynges of mice. J Bacteriol.

[81] Holm MM, Vanlerberg SL, Foley IM, Sledjeski DD, Lafontaine ER (2004). The Moraxella catarrhalis porin-like outer membrane protein CD is an adhesin for human lung cells. Infect Immun.

[82] Murphy TF, Brauer AL, Yuskiw N, Hiltke TJ (2000). Antigenic structure of outer membrane protein E of *Moraxella catarrhalis* and construction and characterization of mutants. Infect Immun.

[83] Adlowitz DG, Sethi S, Cullen P, Adler B, Murphy TF (2005). Human antibody response to outer membrane protein G1a, a lipoprotein of *Moraxella catarrhalis*. Infect Immun.

[84] Ren D, Pichichero ME (2016). Vaccine targets against *Moraxella catarrhalis*. Expert Opin Ther Targets.

[85] Lipski SL, Akimana C, Timpe JM, Wooten RM, Lafontaine ER (2007). The *Moraxella catarrhalis* autotransporter McaP is a conserved surface protein that mediates adherence to human epithelial cells through its N-terminal passenger domain. Infect Immun.

[86] Buskirk SW, Lafontaine ER (2014). *Moraxella catarrhalis* expresses a cardiolipin synthase that impacts adherence to human epithelial cells. J Bacteriol.

[87] de Vries SPW, Bootsma HJ, Hays JP, Hermans PWM (2009). Molecular aspects of *Moraxella catarrhalis* pathogenesis. Microbiol Mol Biol Rev.

[88] Holm MM, Vanlerberg SL, Sledjeski DD, Lafontaine ER (2003). The Hag protein of *Moraxella catarrhalis* strain O35E is associated with adherence to human lung and middle ear cells. Infect Immun.

[89] Tan TT, Nordström T, Forsgren A, Riesbeck K (2005). The respiratory pathogen *Moraxella catarrhalis* adheres to epithelial cells by interacting with fibronectin through ubiquitous surface proteins A1 and A2. J Infect Dis.

[90] Wang W, Pearson MM, Attia AS, Blick RJ, Hansen EJ (2007). A UspA2H-negative variant of *Moraxella catarrhalis* strain O46E has a deletion in a homopolymeric nucleotide repeat common to *uspA2H* genes. Infect Immun.

[91] Myers LE, Yang YP, Du RP, Wang Q, Harkness RE (1998). The transferrin binding protein B of *Moraxella catarrhalis* elicits bactericidal antibodies and is a potential vaccine antigen. Infect Immun.

[92] Luke NR, Howlett AJ, Shao J, Campagnari AA (2004). Expression of type IV pili by *Moraxella catarrhalis* is essential for natural competence and is affected by iron limitation. Infect Immun.

[93] Camacho C, Boratyn GM, Joukov V, Vera Alvarez R, Madden TL (2023). ElasticBLAST: accelerating sequence search via cloud computing. BMC Bioinformatics.

[94] Meier-Kolthoff JP, Auch AF, Klenk H-P, Göker M (2013). Genome sequence-based species delimitation with confidence intervals and improved distance functions. BMC Bioinformatics.

[95] Fassler J, Cooper P (2011). BLAST Glossary.

[96] Pritchard L, Glover RH, Humphris S, Elphinstone JG, Toth IK (2016). Genomics and taxonomy in diagnostics for food security: soft-rotting enterobacterial plant pathogens. Anal Methods.

[97] Kurtz S, Phillippy A, Delcher AL, Smoot M, Shumway M (2004). Versatile and open software for comparing large genomes. Genome Biol.

[98] Meier-Kolthoff JP, Hahnke RL, Petersen J, Scheuner C, Michael V (2014). Complete genome sequence of DSM 30083(T), the type strain (U5/41(T)) of *Escherichia coli*, and a proposal for delineating subspecies in microbial taxonomy. Stand Genomic Sci.

[99] Lefort V, Desper R, Gascuel O (2015). FastME 2.0: a comprehensive, accurate, and fast distance-based phylogeny inference program. Mol Biol Evol.

[100] Kreft L, Botzki A, Coppens F, Vandepoele K, Van Bel M (2017). PhyD3: a phylogenetic tree viewer with extended phyloXML support for functional genomics data visualization. Bioinformatics.

[101] Meier-Kolthoff JP, Göker M (2019). TYGS is an automated high-throughput platform for state-of-the-art genome-based taxonomy. Nat Commun.

[102] Meier-Kolthoff JP, Carbasse JS, Peinado-Olarte RL, Göker M (2022). TYGS and LPSN: a database tandem for fast and reliable genome-based classification and nomenclature of prokaryotes. Nucleic Acids Res.

[103] Meier-Kolthoff JP, Klenk H-P, Göker M (2014). Taxonomic use of DNA G+C content and DNA-DNA hybridization in the genomic age. Int J Syst Evol Microbiol.

[104] Švara A, Sun H, Fei Z, Khan A (2024). Advancing apple genetics research: *Malus coronaria* and *Malus ioensis* genomes and a gene family-based pangenome of native North American apples. DNA Res.

[105] Medini D, Donati C, Tettelin H, Masignani V, Rappuoli R (2005). The microbial pan-genome. Curr Opin Genet Dev.

[106] Kumari K, Rawat V, Shadan A, Sharma PK, Deb S (2023). In-depth genome and pan-genome analysis of a metal-resistant bacterium *Pseudomonas parafulva* OS-1. Front Microbiol.

[107] Segerman B (2012). The genetic integrity of bacterial species: the core genome and the accessory genome, two different stories. Front Cell Infect Microbiol.

[108] Bosi E, Fondi M, Orlandini V, Perrin E, Maida I (2017). The pangenome of (Antarctic) Pseudoalteromonas bacteria: evolutionary and functional insights. BMC Genomics.

[109] Yin Z, Liang J, Zhang M, Chen B, Yu Z (2024). Pan-genome insights into adaptive evolution of bacterial symbionts in mixed host-microbe symbioses represented by human gut microbiota *Bacteroides cellulosilyticus*. Sci Total Environ.

[110] Gaba S, Kumari A, Medema M, Kaushik R (2020). Pan-genome analysis and ancestral state reconstruction of class halobacteria: probability of a new super-order. Sci Rep.

[111] Webb BL, Cox MM, Inman RB (1997). Recombinational DNA repair: the RecF and RecR proteins limit the extension of RecA filaments beyond single-strand DNA gaps. Cell.

[112] Kowalczykowski SC, Dixon DA, Eggleston AK, Lauder SD, Rehrauer WM (1994). Biochemistry of homologous recombination in *Escherichia coli*. Microbiol Rev.

[113] Cox MM (2007). Regulation of bacterial RecA protein function. Crit Rev Biochem Mol Biol.

[114] Lenhart JS, Brandes ER, Schroeder JW, Sorenson RJ, Showalter HD (2014). RecO and RecR are necessary for RecA loading in response to DNA damage and replication fork stress. J Bacteriol.

[115] Chodavarapu S, Kaguni JM (2016). Replication initiation in bacteria. Enzymes.

[116] Kaguni JM (2018). The macromolecular machines that duplicate the *Escherichia coli* chromosome as targets for drug discovery. Antibiotics (Basel).

[117] Lenhart JS, Schroeder JW, Walsh BW, Simmons LA (2012). DNA repair and genome maintenance in *Bacillus subtilis*. Microbiol Mol Biol Rev.

[118] den Hengst CD, Buttner MJ (2008). Redox control in actinobacteria. Biochim Biophys Acta.

[119] Si M, Hu M, Yang M, Peng Z, Li D (2023). Characterization of oxidative stress-induced cgahp, a gene coding for alkyl hydroperoxide reductase, from industrial importance *Corynebacterium glutamicum*. *Biotechnol Lett*.

[120] Bryk R, Griffin P, Nathan C (2000). Peroxynitrite reductase activity of bacterial peroxiredoxins. Nature.

[121] Zhang B, Gu H, Yang Y, Bai H, Zhao C (2019). Molecular mechanisms of AhpC in resistance to oxidative stress in *Burkholderia thailandensis*. Front Microbiol.

[122] Poole LB (2005). Bacterial defenses against oxidants: mechanistic features of cysteine-based peroxidases and their flavoprotein reductases. Arch Biochem Biophys.

[123] Antelmann H, Engelmann S, Schmid R, Hecker M (1996). General and oxidative stress responses in *Bacillus subtilis*: cloning, expression, and mutation of the alkyl hydroperoxide reductase operon. J Bacteriol.

[124] Adams DW, Pereira JM, Stoudmann C, Stutzmann S, Blokesch M (2019). The type IV pilus protein PilU functions as a PilT-dependent retraction ATPase. PLoS Genet.

[125] Real G, Henriques AO (2006). Localization of the *Bacillus subtilis* murB gene within the dcw cluster is important for growth and sporulation. J Bacteriol.

[126] Smith CAS (2006). Structure, function and dynamics in the mur family of bacterial cell wall ligases. J Mol Biol.

[127] Leighton TL, Buensuceso RNC, Howell PL, Burrows LL (2015). Biogenesis of *Pseudomonas aeruginosa* type IV pili and regulation of their function. Environ Microbiol.

[128] Helminen ME, Beach R, Maciver I, Jarosik G, Hansen EJ (1995). Human immune response against outer membrane proteins of *Moraxella* (Branhamella) catarrhalis determined by immunoblotting and enzyme immunoassay. Clin Diagn Lab Immunol.

[129] Garai P, Chandra K, Chakravortty D (2017). Bacterial peptide transporters: messengers of nutrition to virulence. Virulence.

[130] Paulsen IT, Skurray RA (1994). The POT family of transport proteins. Trends Biochem Sci.

[131] Davis GS, Mobley HLT (2005). Contribution of dppA to urease activity in Helicobacter pylori 26695. Helicobacter.

[132] Assmann SM, Armstrong F (1999). Biochemistry and Molecular Biology of Plant Hormones; New Comprehensive Biochemistry.

[133] Weinberg MV, Maier RJ (2007). Peptide transport in *Helicobacter pylori*: roles of dpp and opp systems and evidence for additional peptide transporters. J Bacteriol.

[134] Xu X, Chen J, Huang X, Feng S, Zhang X (2021). The role of a dipeptide transporter in the virulence of human pathogen, *Helicobacter pylori*. Front Microbiol.

[135] Rubio LM, Flores E, Herrero A (1999). Molybdopterin guanine dinucleotide cofactor in *Synechococcus* sp. nitrate reductase: identification of mobA and isolation of a putative moeB gene. FEBS Lett.

[136] Wootton JC, Nicolson RE, Mark Cock J, Walters DE, Burke JF (1991). Enzymes depending on the pterin molybdenum cofactor: sequence families, spectroscopic properties of molybdenum and possible cofactor-binding domains. Biochimica et Biophysica Acta (BBA) - Bioenergetics.

[137] Rajagopalan KV, Neidhardt FC, Ed FC (1996). In Escherichia Coli and Salmonella: Cellular and Molecular Biology.

[138] Amemura M, Makino K, Shinagawa H, Kobayashi A, Nakata A (1985). Nucleotide sequence of the genes involved in phosphate transport and regulation of the phosphate regulon in *Escherichia coli*. J Mol Biol.

[139] Gerdes RG, Rosenberg H (1974). The relationship between the phosphate-binding protein and a regulator gene product from *Escherichia coli*. Biochimica et Biophysica Acta (BBA) - Protein Structure.

[140] Higgins CF, Hiles ID, Whalley K, Jamieson DJ (1985). Nucleotide binding by membrane components of bacterial periplasmic binding protein-dependent transport systems. The EMBO Journal.

[141] Ruan B, Söll D (2005). The bacterial YbaK protein is a Cys-tRNAPro and Cys-tRNA Cys deacylase. J Biol Chem.

[142] Pederick VG, Eijkelkamp BA, Ween MP, Begg SL, Paton JC (2014). Acquisition and role of molybdate in *Pseudomonas aeruginosa*. Appl Environ Microbiol.

[143] Funk CR, Zimniak L, Dowhan W (1992). The pgpa and pgpb genes of *Escherichia coli* are not essential: evidence for a third phosphatidylglycerophosphate phosphatase. J Bacteriol.

[144] Poole K, McKay GA (2003). Iron acquisition and its control in *Pseudomonas aeruginosa*: many roads lead to Rome. Front Biosci.

[145] Liao CH, McCallus DE, Wells JM, Tzean SS, Kang GY (1996). The repb gene required for production of extracellular enzymes and fluorescent siderophores in pseudomonas viridiflava is an analog of the gaca gene of *Pseudomonas syringae*. Can J Microbiol.

[146] Jones MM, Johnson A, Koszelak-Rosenblum M, Kirkham C, Brauer AL (2014). Role of the oligopeptide permease ABC transporter of *Moraxella catarrhalis* in nutrient acquisition and persistence in the respiratory tract. Infect Immun.

[147] Ledwidge R, Blanchard JS (1999). The dual biosynthetic capability of N-acetylornithine aminotransferase in arginine and lysine biosynthesis. Biochemistry.

[148] Hartmann M, Tauch A, Eggeling L, Bathe B, Möckel B (2003). Identification and characterization of the last two unknown genes, dapC and dapF, in the succinylase branch of the L-lysine biosynthesis of *Corynebacterium glutamicum*. J Biotechnol.

[149] Wu Y, Zhang J, Wang B, Zhang Y, Li H (2023). Dissecting the arginine and lysine biosynthetic pathways and their relationship in haloarchaeon Natrinema gari J7-2 via endogenous CRISPR-Cas system-based genome editing. Microbiol Spectr.

[150] Whited AM, Johs A (2015). The interactions of peripheral membrane proteins with biological membranes. Chem Phys Lipids.

[151] Foster TJ, Geoghegan JA, Ganesh VK, Höök MA (2014). Adhesion, invasion and evasion: the many functions of the surface proteins of *Staphylococcus aureus*. Nat Rev Microbiol.

[152] Zwaal RF, Comfurius P, Bevers EM (1998). Lipid-protein interactions in blood coagulation. Biochim Biophys Acta.

[153] Laganowsky A, Reading E, Allison TM, Ulmschneider MB, Degiacomi MT (2014). Membrane proteins bind lipids selectively to modulate their structure and function. Nature.

[154] Muller MP, Jiang T, Sun C, Lihan M, Pant S (2019). Characterization of lipid-protein interactions and lipid-mediated modulation of membrane protein function through molecular simulation. Chem Rev.

[155] Siegel SJ, Weiser JN (2015). Mechanisms of bacterial colonization of the respiratory tract. Annu Rev Microbiol.

[156] Parry BR, Surovtsev IV, Cabeen MT, O’Hern CS, Dufresne ER (2014). The bacterial cytoplasm has glass-like properties and is fluidized by metabolic activity. Cell.

[157] Ebner P, Prax M, Nega M, Koch I, Dube L (2015). Excretion of cytoplasmic proteins (ECP) in *Staphylococcus aureus*. Mol Microbiol.

[158] Ebner P, Götz F (2019). Bacterial Excretion of Cytoplasmic Proteins (ECP): occurrence, mechanism, and function. Trends Microbiol.

[159] Schaar V, de Vries SPW, Perez Vidakovics MLA, Bootsma HJ, Larsson L (2011). Multicomponent *Moraxella catarrhalis* outer membrane vesicles induce an inflammatory response and are internalized by human epithelial cells. Cell Microbiol.

[160] Augustyniak D, Seredyński R, McClean S, Roszkowiak J, Roszniowski B (2018). Virulence factors of *Moraxella catarrhalis* outer membrane vesicles are major targets for cross-reactive antibodies and have adapted during evolution. Sci Rep.

[161] Karner A, Gesslbauer B, Spreitzer A, Almer J, Smidt M (2016). Profiling the membrane and glycosaminoglycan-binding proteomes of *Moraxella catarrhalis*. J Proteome Res.

[162] Borah P, Hazarika S, Chettri A, Sharma D, Deka S (2023). Viral, Parasitic, Bacterial, and Fungal Infections.

[163] Cooper GM (2000). The Central Role of Enzymes as Biological Catalysts.

[164] Edwards KJ, Allen S, Gibson BW, Campagnari AA (2005). Characterization of a cluster of three glycosyltransferase enzymes essential for *Moraxella catarrhalis* lipooligosaccharide assembly. J Bacteriol.

[165] Erridge C, Bennett-Guerrero E, Poxton IR (2002). Structure and function of lipopolysaccharides. Microbes Infect.

[166] Jing W, DeAngelis PL (2003). Analysis of the two active sites of the hyaluronan synthase and the chondroitin synthase of *Pasteurella multocida*. Glycobiology.

[167] Luke-Marshall NR, Edwards KJ, Sauberan S, St Michael F, Vinogradov EV (2013). Characterization of a trifunctional glucosyltransferase essential for *Moraxella catarrhalis* lipooligosaccharide assembly. Glycobiology.

[168] Muñoz-Elías EJ, McKinney JD (2006). Carbon metabolism of intracellular bacteria. Cell Microbiol.

[169] Wang W, Reitzer L, Rasko DA, Pearson MM, Blick RJ (2007). Metabolic analysis of *Moraxella catarrhalis* and the effect of selected in vitro growth conditions on global gene expression. Infect Immun.

[170] Juni E, Heym GA, Avery M (1986). Defined medium for *Moraxella* (Branhamella) catarrhalis. Appl Environ Microbiol.

[171] Schmitz F-J, Beeck A, Perdikouli M, Boos M, Mayer S (2002). Production of BRO beta-lactamases and resistance to complement in European *Moraxella catarrhalis* isolates. J Clin Microbiol.

[172] Esel D, Ay-Altintop Y, Yagmur G, Gokahmetoglu S, Sumerkan B (2007). Evaluation of susceptibility patterns and BRO beta-lactamase types among clinical isolates of *Moraxella catarrhalis*. Clin Microbiol Infect.

[173] Bootsma HJ, Aerts PC, Posthuma G, Harmsen T, Verhoef J (1999). *Moraxella* (Branhamella)catarrhalis BRO β-lactamase: a lipoprotein of gram-positive origin?. J Bacteriol.

[174] Adlowitz DG, Kirkham C, Sethi S, Murphy TF (2006). Human serum and mucosal antibody responses to outer membrane protein G1b of *Moraxella catarrhalis* in chronic obstructive pulmonary disease. FEMS Immunol Med Microbiol.

[175] Assalkhou R, Balasingham S, Collins RF, Frye SA, Davidsen T (2007). The outer membrane secretin PilQ from *Neisseria meningitidis* binds DNA. Microbiology.

[176] Forest KT, Satyshur KA, Worzalla GA, Hansen JK, Herdendorf TJ (2004). The pilus-retraction protein PilT: ultrastructure of the biological assembly. Acta Crystallogr D Biol Crystallogr.

