# Supplementary material for: A global perspective on the genomics of Moraxella catarrhalis
Source: Microb Genom. 2025 Aug 22;11(8):001488. doi: 10.1099/mgen.0.001488 (PMC12373483; doi:10.1099/mgen.0.001488)
Supplement: Uncited Fig. S1. [file mgen-11-01488-s001.pdf]

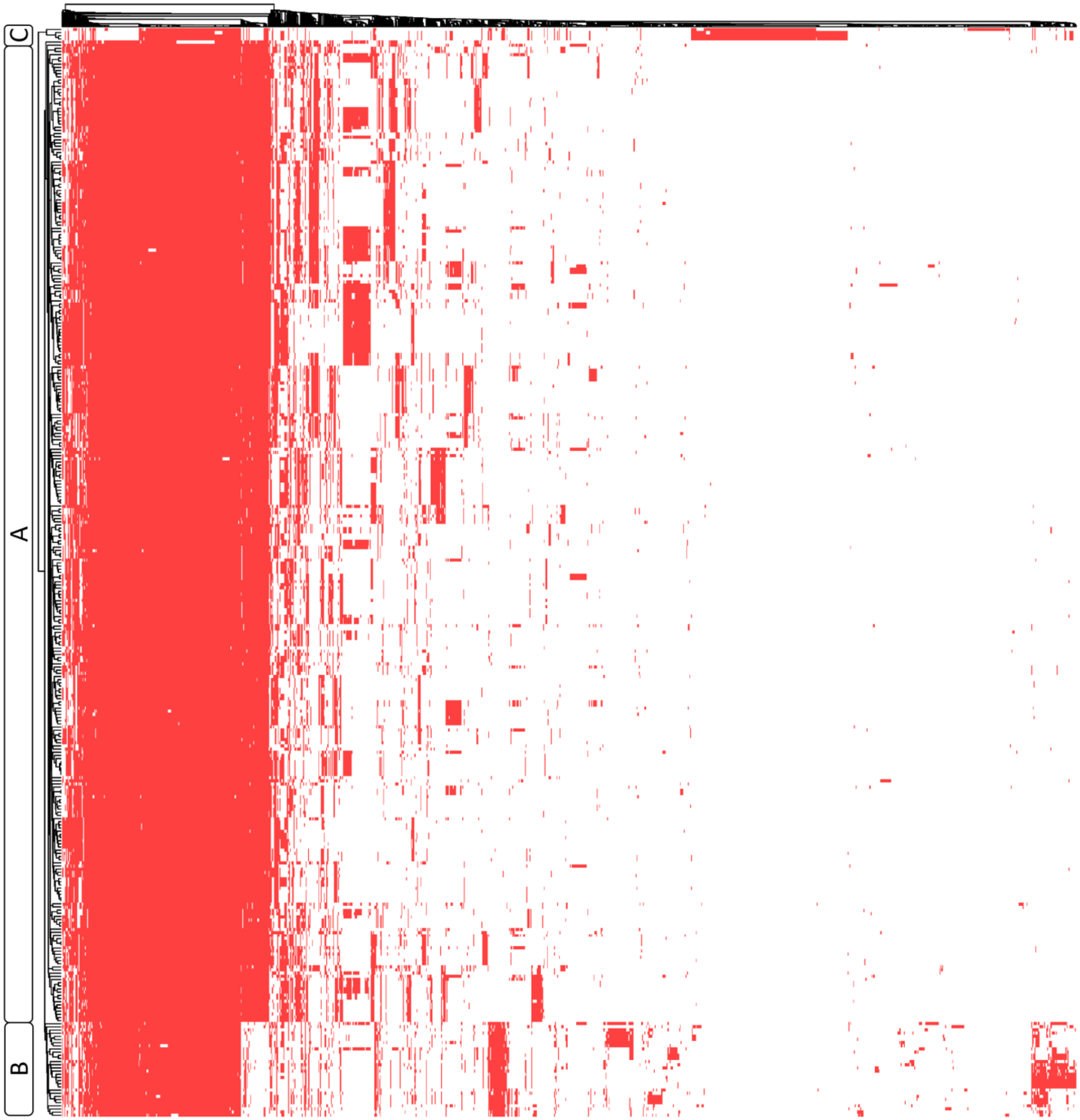

Figure S1. Accessory genome variability among Phylogroups A, B, and C. The heatmap shows the presence/absence of accessory genes across the analyzed genomes and is accompanied by a dendrogram clustering the strains based on the similarity of their accessory gene content. Genes present are marked in red. Letters A, B, and C indicate the classification of each strain within the three phylogroups identified through phylogenetic analysis.

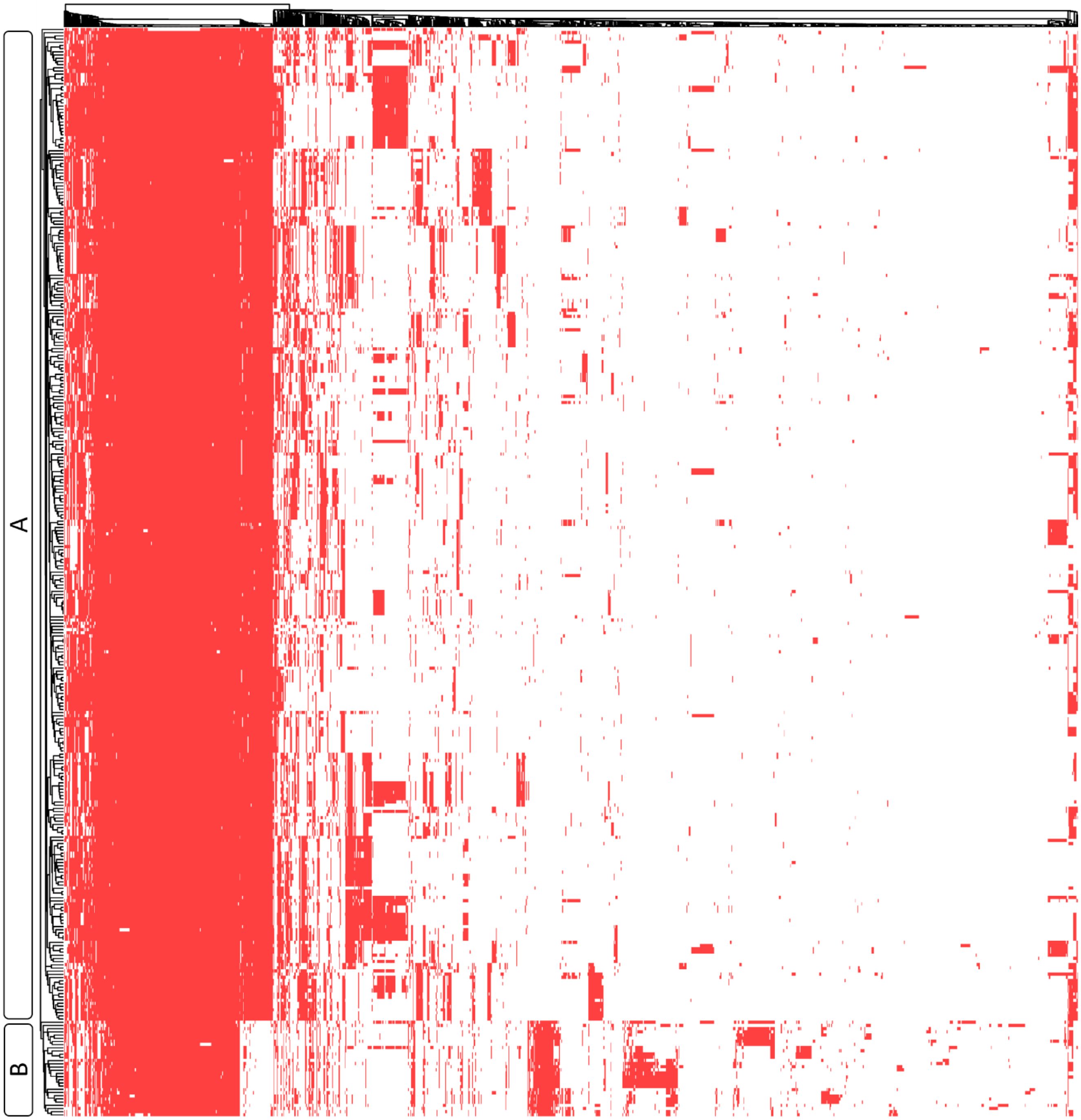

Figure S2. Accessory genome variability among Phylogroups A and B. The heatmap shows the presence/absence of accessory genes across the analyzed genomes and is accompanied by a dendrogram clustering the strains based on the similarity of their accessory gene content. Genes present are marked in red. Letters A and B indicate the classification of each strain within the three phylogroups identified through phylogenetic analysis.
